# Micelles Formed by Polypeptide Containing Polymers Synthesized Via *N*-Carboxy Anhydrides and Their Application for Cancer Treatment

**DOI:** 10.3390/polym9060208

**Published:** 2017-06-04

**Authors:** Dimitrios Skoulas, Panagiotis Christakopoulos, Dimitra Stavroulaki, Konstantinos Santorinaios, Varvara Athanasiou, Hermis Iatrou

**Affiliations:** Department of Chemistry, University of Athens, Panepistimiopolis, Zografou, Athens 15771, Greece; dskoulas@chem.uoa.gr (D.Sk.); panchristak@chem.uoa.gr (P.C.); stavrulakidim@yahoo.gr (D.St.); santor.g7@gmail.com (K.S.); barbara92athanasiou@gmail.com (V.A.)

**Keywords:** polypeptides, micelles, drug delivery, gene delivery, biomaterials, ring-opening polymerization of *n*-carboxy anhydrides, cancer, biomedical applications

## Abstract

The development of multifunctional polymeric materials for biological applications is mainly guided by the goal of achieving the encapsulation of pharmaceutical compounds through a self-assembly process to form nanoconstructs that control the biodistribution of the active compounds, and therefore minimize systemic side effects. Micelles are formed from amphiphilic polymers in a selective solvent. In biological applications, micelles are formed in water, and their cores are loaded with hydrophobic pharmaceutics, where they are solubilized and are usually delivered through the blood compartment. Even though a large number of polymeric materials that form nanocarrier delivery systems has been investigated, a surprisingly small subset of these technologies has demonstrated potentially curative preclinical results, and fewer have progressed towards commercialization. One of the most promising classes of polymeric materials for drug delivery applications is polypeptides, which combine the properties of the conventional polymers with the 3D structure of natural proteins, i.e., *α*-helices and *β*-sheets. In this article, the synthetic pathways followed to develop well-defined polymeric micelles based on polypeptides prepared through ring-opening polymerization (ROP) of *N*-carboxy anhydrides are reviewed. Among these works, we focus on studies performed on micellar delivery systems to treat cancer. The review is limited to systems presented from 2000–2017.

## 1. Introduction

In nature, complex and diverse structures are constructed from a finite choice of building units, such as the amino acids for the synthesis of proteins. Even higher in complexity is the cell, which is produced in large variety in nature to address specific function mostly through the concerted action of perfectly defined proteins having the required conformation. Contrary to macromolecules produced in nature, synthetic polymers can be obtained through “living” polymerization techniques [1,2] from a very large variety of monomers, in miscellaneous architectures, including not only linear but also branched systems. However, none of these structures displays the sophistication and complexity of those based on the combination of 20 natural amino acids. Today, the ultimate challenge of synthetic polymer chemists is to prepare polymers that can self-organize through purely physical forces, simulating the folding of peptides in proteins, to achieve nanoconstructs (NCs) with the appropriate functionality. The cornerstone of this strategy is the chemical structure of the synthetic polymers that should carry all the information to direct the self-assembly process, as in the case of protein primary structure. The organization of the synthetic molecules has to be so advanced that they will be able to self-assemble from a few angstroms up to a few microns. The only materials that can be organized at such a hierarchical level, besides natural proteins, are polypeptides. Polypeptides are composed of repeating amino acids, connected by an amide bond and present 3D structures such as *α*-helix, *β*-sheet, like natural proteins. Mimicking natural biopolymers with synthetic polypeptides has both advantages and limitations. Although synthetic polypeptides do not present the biological functionality of the natural proteins, they have the advantage of being composed of much simpler components than natural proteins, can be prepared in large quantities, are usually biocompatible and most importantly, presents the 3D structures of the natural proteins. Due to these properties, synthetic polypeptides are used as the building materials of nanoconstructs for drug delivery applications.

Cancer treatment remains a major challenge in medicine. In addition to the rise in cancer incidences, another important fact is the relatively modest progress in the development of new drugs and advances in treatment modalities, as illustrated by the persistent mortality rates still observed for some types of cancers. Pharmaceutical scientists are trying to shift from traditional to novel drug delivery systems (DDS) by applying nanotechnology and especially polymeric carriers to medicine, in order to maximize therapeutic benefits drugs, genes and proteins should be delivered with enhanced therapeutic efficacy, at reduced doses and frequency and with fewer side effects. Although research to identify more efficient drugs is rapidly advancing, the discovery of novel materials with the required functionality of targeted and efficient drug delivery to cancer cells is not progressing at the same rate.

During the past two decades, there has been tremendous progress in the design of materials at the nanoscale level that has paved the way for a new category of healthcare technologies generally termed nanomedicine. Nanomedicine involves manipulating, engineering, and exploiting different material properties at the nanometer level in order to design products that can improve current technologies for biomedical applications. While traditional therapeutic agents have allowed for very little control in terms of their distribution in the body and clearing times, engineering at the nanoscale level has allowed for significant advances in optimizing the biocompatibility, biodistribution, and pharmacokinetics of various medical technologies. Among the materials used to design and produce therapeutics for nanomedicine applications, polymeric materials have dominated the nanomedicine field. This is due to their high functionality and the possibility to manipulate their properties by combining different materials in a wide variety of macromolecular architectures. Such architectures eventually direct the self-organization of the materials into nanostructures that possess the desired properties for effective drug/gene encapsulation as well as selective and controlled delivery.

The development of novel polymeric materials is guided by the goal of improving patient survival and quality of life by reducing chemotherapy side effects. Intravenous delivery has been shown to be one of the most effective ways to target cancer cells, since it can target not only the located cancer tissues but also circulating tumor cells which are responsible for metastasis. Effective drug and gene delivery through the systemic route to the target site carriers requires that several extra- and intracellular barriers are avoided.

In cancer treatment, the nanomaterials locate their target by passive targeting via leaky tumor vasculature and the enhanced permeability retention (EPR) effect [3], by active targeting by conjugation of a chemical moiety overexpressed in cancer cells (monoclonal antibody, folic acid for cancer treatment) [4], by introducing stimuli-responsive triggering for the release of the payload under environmental conditions that can be found only in cancer tissues, or by the combination of all the above methods. Typical stimuli include pH, temperature, light, magnetic field, electrolytes or glucose [5,6]. In order for these nanoparticles to find and accumulate at their target cells through the systemic route they need to overcome several extra- and intracellular barriers by fulfilling several major prerequisites: (a) their outer periphery must be inert in order to avoid the activation of the reticuloendothelial system (RES) so that they can circulate in the blood compartment for prolonged periods of time, increasing the probability of reaching the pathological cells, (b) their dimensions should be close to 100 nm in order to escape the blood compartment and locate the pathological cells, (c) after internalization in the pathological cells, the nanoparticles should be able to escape the endosome, otherwise exocytosis could occur and prevent drug release into the cytoplasm, (d) the drugs or genes should present cytoplasmic stability and (e), in order for genes or other large molecules to be internalized into the nucleus, they should be recognized by the nuclear pore complex. It is obvious that due to the large number of barriers, nanoparticles should combine a significant number of functionalities in order to bypass them. However, to date, the functionality of the nanoparticles presented is limited, and therefore the mean accumulation of intravenously administered nanocarriers to cancer tissues is very low, close to 1% [7].

Micelles are nanoparticles composed of a hydrophobic core and a hydrophilic shell. Hydrophobic pharmaceutics are usually dissolved and encapsulated within the hydrophobic core through hydrophobic interactions, which otherwise could not be delivered in the aqueous environment of the body.

**Figure 1:**
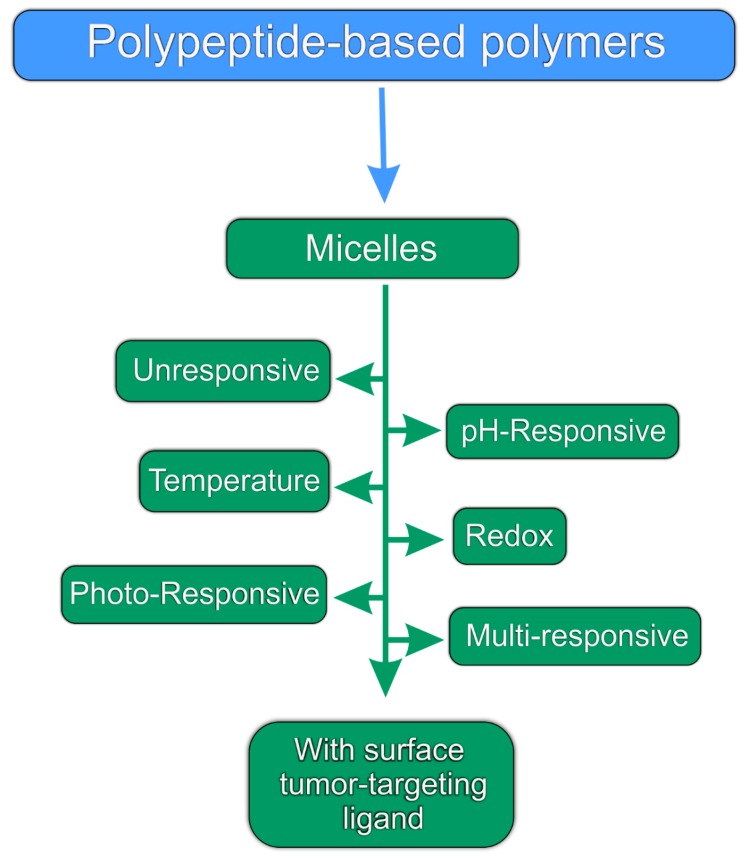
Structure of the smart aggregates formed by polypeptide-containing polymers presented in this review.

In this review, we highlight the recent progress in the synthesis of polypeptide-containing polymers that form micelles, and particularly those that have been synthesized utilizing the ring opening polymerization (ROP) of *N*-carboxy anhydrides (NCAs) as the monomers, in a living/controlled polymerization process. The scope of the review is also focused on the use of these materials in publications between 2000–2017 for the encapsulation and delivery of anticancer agents (Figure 1).

## 2. Unresponsive Micelles

Polypeptide-based materials present unique properties associated with the 3D secondary structure, such as *α*-helix and *β*-sheet. This property drives the self-assembly to higher orders, and depending on the environmental conditions such as pH or temperature can change, leading to disruption of aggregates and release of the cargo. Although most of the novel polypeptide-based materials that form micelles present stimuli-responsive characteristics to deliver the cargo under tumor tissue conditions in a targeted way, some of them do not present responsiveness and are based on passive targeted delivery through the enhanced permeability and retention (EPR) mechanism.

**Figure 2:**
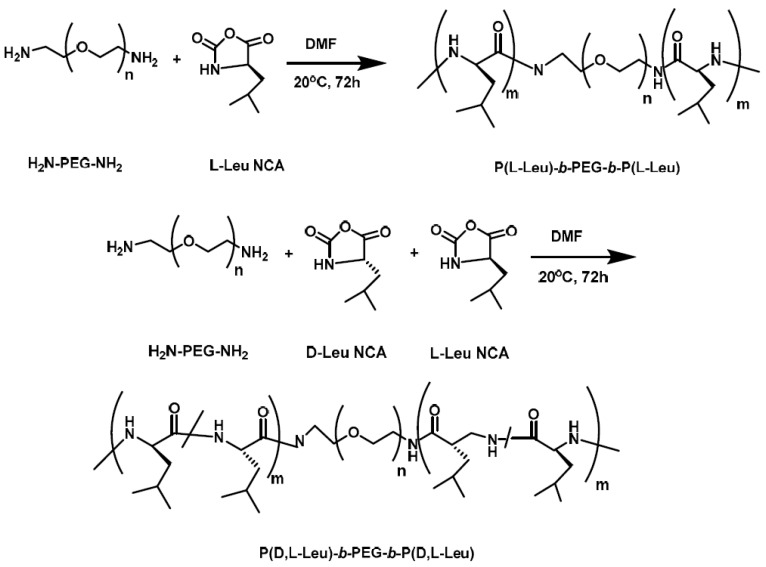
Synthesic approach for the synthesis of P(l–Leu)–*b*–PEG–*b*–P(l–Leu) and P(d,l–Leu)-*b*-PEG–*b*–P(d,l–Leu) [8].

In order to examine micellar properties and antitumor potential drug delivery efficiency, Ding et al. [8] synthesized two kinds of triblock copolymers of the type A–*b*–B–*b*–A, where B is poly(ethylene oxide) (PEO or PEG) and A is polyleucine (PLeu). Both contain PEG blocks forming the outer shell of the micelle, while the second block is either levorotatory polyleucine or racemic polyleucine comprising the hydrophobic core. *α*,*ω*-Diamino end-functionalized poly(ethylene oxide) (NH_2_–PEG–NH_2_ or NH_2_–PEO–NH_2_) was used as the macroinitiator for the ROP of l-leucine NCA and the same macroinitiator used for the ROP of equimolar amounts of d-leucine NCA and l-leucine NCA (Figure 2). Micelles were prepared by dissolving the polypeptides in DMF and addition of PBS. Subsequently, dialysis occurred against PBS for 48 hrs. For the encapsulation of Doxorubicin (DOX), the most common anticancer drug, the drug and the copolymer were dissolved in DMF and after stirring for 3 h deionized water and PBS were added dropwise. The solution was dialyzed against deionized water, followed by lyophilization. The polymer with levorotatory polyleucine had a higher critical micellar concentration and a higher diameter as well as quicker DOX release in PBS due to its strong *α*-helical secondary conformation. The polymer with racemic polyleucine had a higher drug loading capacity (DLC) and drug loading efficiency (DLE) because of its more compact micellar core.

In another work, Liu et al. [9] described the synthesis of a bio-inspired amphiphilic diblock copolymer as a drug delivery system (DDS) for cancer treatment. They took advantage of the biocompatibility and biodegrability of the monomers to synthesize poly(*γ*-benzyl–l-glutamate)–*b*-poly(2-methacryloyloxyethyl phosphorylcholine) (PBLG–*b*–PMPC) copolymer. The synthesis of the amphiphilic PBLG–*b*–PMPC copolymer occurred via a two-step procedure. The heterofunctional initiator, *N*-(2-aminoethyl)-2-bromo-2-methylpropanamide, was initially synthesized. The synthetic procedure involved the reaction of *N*-Boc-ethylenediamine with 2-bromoisobutyryl bromide in the presence of triethylamine. The primary amine of the initiator was used for the ring-opening polymerization (ROP) of the *N*-carboxy anhydride of BLG (BLG–NCA), and the activated bromide was used as the initiating species for the atom transfer radical polymerization (ATRP) of MPC in the presence of CuBr/bpy as the catalyst, to afford the diblock copolymer. Nuclear magnetic resonance (^1^H NMR) was used to estimate the successful synthesis of the PBLG–Br macroinitiator. Size exclusion chromatography (SEC) was used to calculate the polydispersity value (PDI) of the polymer. ^1^H NMR analysis proved the successful synthesis of PBLG–*b*–PMPC copolymer. SEC analysis was used for the analysis of the final copolymer PBLG–*b*–PMPC. This polymer self-assembled into micelles with PBLG as the hydrophobic core and PMC as the hydrophilic shell. DOX was encapsulated into the micelle core through *π–π* hydrophobic interactions with the aromatic rings of the protective groups of PBLG. Hydrophilic DOX·HCl was neutralized by triethylamine in dimethylformamide (DMF) to remove HCl and for the conversion to hydrophobic DOX. DOX, along with PBLG–*b*–PMPC, were then dissolved in CH_3_OH and DMF followed by addition of phosphate-buffered saline (PBS) solution. The mixture was dialyzed against PBS to remove the unencapsulated drugs. The above synthetic route took place in the dark. The DOX-loaded micelles were lyophilized, and the drug loading content (DLC) was determined by UV spectroscopy. Dynamic light scattering (DLS) analysis estimated that the diameter of the DOX-loaded micelles was about 70 nm, larger than that of DOX-free micelles (56nm), proving the successful dissolution of DOX into the PBLG core. The structure of DOX-loaded PBLG-*b*-PMPC micelles was obtained by transmission electron microscopy (TEM) analysis which indicated spherical morphology independent of drug loading.

**Figure 3:**
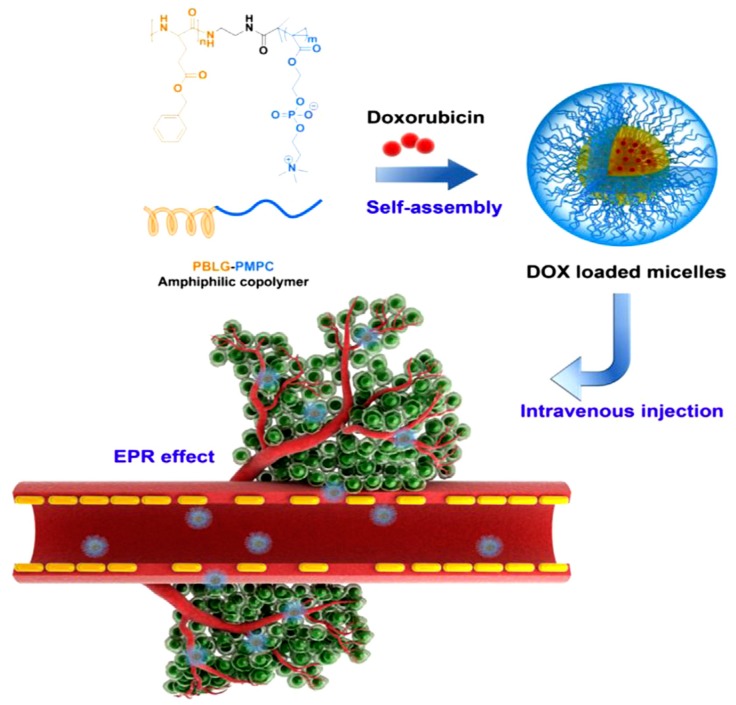
Self-assembly, drug loading and tumor accumulation of PBLG–*b*–PMPC polymeric micelles [9].

Subsequently, in vitro and in vivo cellular uptake and cytotoxicity studies of both DOX-loaded micelles and free DOX indicated the enhanced antitumor efficacy of the drug-loaded micelles compared with the free DOX, confirming the synthesis of a novel nano-scale micellar anticancer drug carrier (Figure 3).

## 3. pH-responsive Micelles

Numerous studies have been conducted on the role of pH-responsive materials as DDS, since pH is an important stimulus, especially concerning biological applications and drug delivery processes. Many ionisable polypeptides have been used as potential candidates, due to their conformational, size and solubility changes, which originate from changes in ionisation state upon pH variations. Moreover, the presence of groups which exhibit ionisation state changes depending on pH lead to the control of drug release as a function of time.

In 2008, Yokoyama et al. [10] reported the synthesis of adriamycin-conjugated poly(ethylene glycol)–*b*–poly(aspartic acid) block copolymers (PEG–*b*–P[Asp(ADR)]). Targeted drug delivery was achieved by the appropriate composition of the block copolymer-drug conjugates. The synthesis of PEG–*b*–P[Asp(ADR)] was achieved using *α*-methyl-*ω*-amino poly(ethylene oxide) as macroinitiator for the polymerization of *β*-benzyl-l-aspartate *N*-carboxy anhydride (BLA–NCA) in order to obtain PEG–*b*–poly(*β*-benzyl-l-aspartate) block copolymer (PEG–*b*–PBLA). The benzyl protective groups were cleaved by alkaline hydrolysis to obtain PEG–*b*–PAsp. Adriamycin (ADR) was conjugated to PAsp block by amide bond formation between the amino group of the ADR molecule and the carboxyl group of the aspartic acid residue in the presence of 1-ethyl-3-(3-dimethylaminopropyl) carbodiimide. Eight conjugates with various PEG and PAsp chain lengths were obtained varying between 1000 and 12,000 for PEG and 900 and 8700 for PAsp. These conjugates formed micellar structures ranging from approximately 10 to 100 nm in diameter, as obtained by dynamic laser light scattering measurements.

Amphiphilic poly(*β*-benzyl-l-aspartate)–*b*–poly(vinylpyrrolidone), PAsp(OBzl)–*b*–PVP, copolymers were synthesized by a combination of ATRP and ROP polymerizations. The synthetic approach involved the polymerization of *β*-benzyl-l-aspartate *N*-carboxy anhydride (Asp(OBzl)–NCA) initiated by the amino-terminated PVP (PVP–NH_2_) [11]. The obtained diblock copolymers were investigated for their micellar behavior. From DLS measurements, it was found that the mean hydrodynamic diameters were in the range of 40–60 nm, and the size distribution indicated a narrow and monodisperse pattern. TEM images of micelles formed from PAsp(OBzl)_82_–*b*–PVP_55_ diblock copolymers showed well-ordered micelles. The critical micelle concentration (CMC) was also obtained by a fluorescence probe technique, and it was found that by decreasing the length of the hydrophobic PAsp(OBzl) segment, the CMC increased. Drug release behavior was also studied using prednisone acetate (PAC) as a model drug to be entrapped into the hydrophobic core of amphiphilic block copolymer micelles. The drug loading efficiency (DLE) and drug loading content (DLC) increased with the drug to polymer weight ratio. The pH dependent PAC release profile showed that the amount and rate of its release from the micelles at pH 7.4 were much lower when compared to that at pH 2.1.

In 2011, Howard et al. [12] synthesized poly(ethylene glycol)-poly(*β*-dexamethasone-l-aspartate) [PEG–*b*–P(Asp–Est–DEX)] block copolymers for the targeted release of dexamethasone (DEX) in tumors, by conjugation of DEX to aspartic acid groups using hydrazone, ester, or hydrazone-ester dual linkers. The synthesis of the polymer was conducted using *α*-methoxy-ω-amino-poly(ethylene glycol) (PEG–NH_2_) as macroinitiator of the BLA–NCA. In a subsequent procedure, deprotection of the benzyl ester groups using PEG–*b*–PBLA was achieved by dissolving it in aqueous NaOH solution. The solution was dialyzed against deionized water until NaOH was removed completely to afford PEG–poly(l-aspartatic acid) [PEG–*b*–PAsp].

Two different DEX conjugation products were synthesized. In the first product, DEX was conjugated to PEG–*b*–P(Asp) through esterification using *N*,*N*’-diisopropylcarbodiimide (DIC) and 4-(dimethylamino)pyridine (DMAP), to afford [PEG–P(Asp–Est–DEX)], EST-M. In addition to this material, PEG–*b*–PBLA bearing DEX through acid-labile hydrazone groups was also synthesized. The hydrazone groups were introduced through aminolysis reactions of the benzyl groups of the polypeptide PEG–*b*–PBLA using anhydrous hydrazine, to afford PEG–*b*–poly(aspartate hydrazide) [PEG–*b*–P(Asp–Hyd)]. PEG–*b*–p(Asp–Hyd) block copolymers were subsequently reacted with three ketonic acids (4-acetylbutyric acid (ABA), 6-oxoheptanoic acid (OHA), 7-oxooctanoic acid (OOA)), followed by reaction with the hydroxyl group of DEX in the presence of DIC and DMAP to form the ester. The ketonic acids provided spacers of 3, 4 and 5 methylene groups (“X”) between PEG–*b*–P(Asp–Hyd) and DEX in the final products [PEG–*b*–P(Asp–Hyd–X–Est–DEX). ^1^H NMR and SEC measurements confirmed the success of synthesis, and the molecular weight distribution of the block copolymers was rather high, with a polydispersity index of 1.3.

The micelles were formed by using either reconstitution or freeze-drying methods. In the first method, the drug-conjugated polymer was dissolved in aqueous solutions followed by sonication. Block copolymers were dissolved in acetonitrile (ACN) and diluted with deionized water in the freeze-drying method. The block copolymer solutions were freeze-dried, the solid powders containing the micelles were reconstituted in aqueous solutions and all micelles were filtered. The micelles resulting from the materials with the spacers exhibited low polydispersity and were smaller than 100 nm: ester (EST-M) (85.74 nm), hydrazone–ABA–ester (ABA-M) (61.50 nm), hydrazone–OHA–ester (OHA–M) (43.82 nm), and hydrazone–OOA–ester (OOA–M) (37.84 nm). Drug release patterns from the micelles formed by EST-M indicated that the micelles were unstable at pH 7.4, but more stable at pH 5.0, i.e., the release was faster at higher pH values. On the contrary, in all cases of the hydrazine linkers (ABA–M, OHA–M, and OOAM), it was found that DEX release from the micelles was suppressed at pH 7.4 and accelerated at pH 5.0. The polymer micelles with hydrazone–ester dual linkers may be stable in blood and release higher quantities of DEX in acidic tumor tissues. At pH 5.0, DEX release was reduced upon increase in the chain length of the spacer. It should be noted that DEX release was less affected by the spacer at pH 7.4. To inhibit DEX release at pH 7.4, ketonic acids longer than OOA were used, but the block copolymers precipitated.

**Figure 4:**
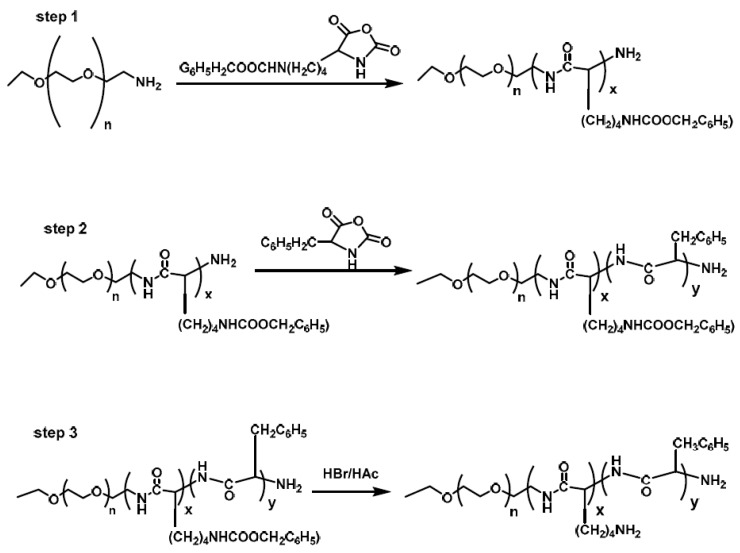
Synthesis of the triblock polypeptide copolymer poly(ethylene glycol)–*b*–poly(l-lysine)-*b*-poly(l-phenylalanine).

Li et al. [13] synthesized a hybrid polypeptide micelle based on poly(ethylene glycol)–*b*–poly(l-lysine)–*b*–poly(l-phenylalanine) (PEG*–b–*PLL–*b*–PPhe) with the sequential ROP of the NCAs of ε-benzyloxycarbonyl–l-lysine (ZLL–NCA) and L-phenylalanine. For the synthesis of the triblock copolymers, PEG–NH_2_ was used as the macroinitiator. The deprotection of the benzyl groups was accomplished with the addition of trifluoroacetic acid and hydrobromic acid (HBr) in acetic acid (HAc) (Figure 4). The hybrid terpolymers were examined in order to find the CMC. It was found that by increasing the hydrophilic block, CMC values decreased. TEM measurements showed that the micelles had a spherical shape and diameters of approximately 25 nm, while the mean size of the micelle was approximately 45 nm. Additional confirmation for the micellization behavior was contributed by ^1^HNMR. It was found that after the freeze-drying, the proton signals in the benzene groups of Phe units had almost disappeared, indicating a core/shell micellar structure. Due to the amino groups on the side chains of the PLL segment, the terpolymers were pH-sensitive. It was found that upon increasing the pH, the hydrodynamic diameter of the PEG–*b*–PLL–*b*–PPhe micelles decreased. Furthermore, agarose gel retardation assay showed that the polymers with higher PLL ratio condense DNA more easily. Furthermore, the study of ζ potential measurements showed that the polymers could bind DNA to form nanoparticles exhibiting positive surface charges, suggesting that PEG–*b*–PLL–*b*–PPhe/DNA complexes can be used for gene transfection. In addition to the condensation of DNA for the formation of anticancer nanoparticles, DOX was encapsulated inside the micelles formed by the amphiphilic polymer via the nanoprecipitation methodology. The in vitro drug release study of DOX revealed negligible release of the drug at neutral and alkali pH, while in acidic environment a two-phase drug release occurred.

The synthesis of amphiphilic dendritic poly(glutamic acid)–*b*–polyphenylalanine copolymers was achieved with generation 3 dendritic poly(glutamic acid) (PLGA) as the macroinitiator in the ROP of Phe–NCA [14]. The self-assembly of the dendritic PLGA–*b*–PPhe copolymer micelles resulted from the large amount of peripheral carboxyl groups of dendritic PLGA and the hydrophobic PPhe segment. Hydrophobic DOX was loaded in the core of core–shell structured micelles. The CMC of the micelles was tested using pyrene as a fluorescent probe, while the size distributions of the blank and DOX-loaded micelles were investigated by DLS. TEM was also employed to investigate the structure of both blank and drug loaded micelles and it was found with both methods that the micelles were monodisperse spherical particles with sizes ranging from 80 to 100 nm.

The release profile of the DOX-loaded micelles was carried out in PBS solution (pH 7.4) at 37 °C. Both free DOX hydrochloride and hydrophobic DOX were used as controls. No release effect was observed in free DOX, while it was found that more than 90% of the DOX∙HCl was released within 2 h. In contrast, less than 20% DOX was released in the micellar system over the same period. The sustaining release time of the DOX through the micelle was more than 60 h. The biocompatibility of the self-assembled micelles was examined in NIH/3T3 cells with the CCK-8 assay, which measures cytotoxicity via concentration and time. High cell viability was found for the self-assembled blank micelles at 12, 24 and 48 h.

Ding et al. [15] reported the synthesis of five pH-responsive alkyne–poly(2-aminoethyl methacrylate)–*g*–poly(l-glutamic acid) (alkyne–PAMA–*g*–PLGA) comb copolymers through the ROP of BLG–NCA and the deprotection of the benzyl group. The synthetic approach first involved the synthesis of alkyne–poly(2-aminoethyl methacrylate hydrochloride) (alkyne–PAMA) by the atom transfer radical polymerization (ATRP) of 2-aminoethyl methacrylate hydrochloride (AMA). Alkyne-poly(2-aminoethyl methacrylate)–*g*–poly(*γ*-benzyl–l-glutamate) (alkyne–PAMA–*g*–PBLG) was then synthesized through ROP of BLG–NCA using the amine groups of alkyne–PAMA as macroinitiator, followed by deprotection of the PBLG block to provide alkyne-PAMA–*g*–PLGA. Finally, rhodamine-B functionalized block copolymer (RhB–PAMA–*g*–PLGA) was synthesized by conjugation the rhodamine B-azide (RhB–N_3_) onto alkyne–PAMA–*g*–PLGA by click chemistry. The micelles were loaded with DOX through the nanoprecipitation methodology, to obtain the DOX-loaded nanoparticles.

Nanoparticle sizes were determined by DLS in PBS at pH 6.8, 7.4, and 8.0. The comb copolymers alkyne–PAMA_12_–*g*–PLGA_13_, alkyne–PAMA_14_–*g*–PLGA_12_, alkyne–PAMA_16_–*g*–PLGA_12_, alkyne–PAMA_16_–*g*–PLGA_28_, alkyne–PAMA_16_–*g*–PLGA_52_, all assembled into micelles, with the exception of alkyne–PAMA_16_–*g*–PLGA_12_ which formed vesicles in PBS at pH 7.4. Self-assembly of the alkyne–PAMA–*g*–PLGA comb copolymers into micellar or vesicular nanoparticles with hydrodynamic radii (Rh) values between 41.9 and 268 nm, depending on the copolymer composition and pH values (pH 6.8, 7.4, and 8.0), confirmed that nanoparticle stability depends on the molecular weight of both the PAMA backbone as well as the PLGA side chain. Both the increase of the hydrophobic segment content and the decrease of hydrophilic moiety content resulted in a decrease in the CAC value. The size of the nanoparticles could also be altered by the comb copolymer composition.

Comparison of the drug loading capabilities of nanoparticles revealed that higher DLC% and DLE% resulted from the higher DP of the PLGA side chains. The DOX release rate was also a result of the electrostatic interaction between the amine of DOX and the carboxyl groups of PLGA. The drug release was pH-dependent, and the release rate increased in the order pH 6.8 > pH 7.4 > pH 8.0. At acidic pH, DOX release increased, likely because of the protonation of nanoparticle carboxylic groups, which limited the interaction between nanoparticle and DOX.

In 2012, Kataoka group [16] reported the integration of the homopolymer poly{*N*’-[*N*-(2-aminoethyl)-2-aminoehtyl]aspartamide} [PAsp(DET)] into the copolymer PEG–*b*–PAsp(DET) in order to formulate a micelle that presented an increased cell transfection efficiency of pDNA in fibrotic pancreatic tumor cells. PAsp(DET) homopolymer synthesized by ROP polymerization of monomer BLA–NCA using two kind of initiators, *a*-methoxy-*ω*-amino-poly (ethylene glycol) as macroinitiator producing PEG–*b*–PBLA and also *n*-butylamine producing PBLA. Polymerization was followed by aminolysis reaction introducing diethylenetriamine molecules into the side chain of PBLA. ^1^H NMR and SEC analysis confirmed the successful synthesis. It was also found that all synthesized polymers had a narrow unimodal molecular weight distribution. The polyplex micelles were formed by dissolving separately PEG–*b*–PAsp(DET) and PAsp(DET) at pH 7.4. Mixture of these stock solutions at varying PEG–*b*–PAsp(DET)/PAsp(DET) ratios was added to pDNA solution for complexation at varying N/P ratios (residual molar ratio of total amino groups in PAsp(DET) and PEG–*b*–PAsp(DET) to phosphate groups in pDNA). The size and polydispersity index (PDI) of polyplex micelles were determined by DLS and TEM. All samples containing only the PEG–*b*–PAsp(DET) had diameters from 60 to 100 nm with unimodal size distributions and low PDI from 0.1 to 0.2. The polyplex which formed from PAsp(DET) and pDNA had a larger size, over 1000 nm, at a critical *N*/*P* range of 1.5–2. Over this critical *N*/*P* range (*N*/*P* ≥ 2), the polyplex formed from PAsp(DET) and pDNA possessed comparable size of approximately 75 nm with unimodal size distribution. The zeta potential determined through laser-doppler electrophoresis showed negative net charge of pDNA in all complexes, and was approximately neutralized at a critical N/P of 1.5–2, which is consistent with the protonation degree (53%) of amino groups in PAsp(DET) at pH 7.4. It was found that the polyplex micelles resulting from the mixture PEG–*b*–PAsp(DET)/PAsp(DET) and pDNA present uniform rod-shaped particles, suggesting that the DNA strand is packaged into the rod-shaped bundle through a regular folding behavior, whereas the polyplex micelle resulting from PAsp(DET) and pDNA without PEG surface adopted a completely collapsed spherical configuration. This difference shows the important role of the PEG chains in mediating pDNA packaging. It was found that the PEG–*b*–PAsp(DET)/PAsp(DET) ratio for the optimum transfection efficiency was between 50 and 70. These novel polypeptide-based micelles are promising drug carriers for the treatment of pancreatic cancer.

In 2012, Akter et al. [17] reported the synthesis of poly(ethylene glycol)–*b*–poly(*b*-hydrazide-l-aspartate) [PEG–*b*–PHYD] micelle block copolymers for the controlled delivery of a glycolytic enzyme inhibitor, 3-(3-pyridinyl)-1-(4-pyridinyl)-2-propen-1-one (3PO). 3PO was conjugated to these block copolymers through an acid-labile hydrazone bond. Initially, PEG–*b*–PBLA was synthesized through the ROP of BLA–NCA using PEG–NH_2_ as the initiator, followed by modification of the carboxyl groups by various functional groups either by conjugation or aminolysis reactions. For conjugation reactions, all benzyl esters on PEG–*b*–PBLA were deprotected with NaOH in advance to prepare PEG–*b*–PAsp block copolymers. Characterization by ^1^H NMR as well as SEC showed the high purity of the polymers. For aminolysis reactions, anhydrous hydrazine was reacted with dry PEG–*b*–PBLA to afford PEG–*b*–PHYD block copolymers, as confirmed by ^1^H NMR. 3PO as well as the aliphatic compounds 2-hexanone, 4-acetylbutyric acid and 4-oxohexanoic acid were then chemically conjugated with PEG–*b*–PHYD through acid-labile hydrazone bond by the reaction of the ketone groups of the low molecular substances with the hydrazide groups of block copolymers. In addition, PEG–*b*–PAsp was conjugated with 3-bromobiphenyl, 1,3-diphenyl-2-bromo-propane and chloromethyl phenyl sulfoxide to give PEG–*b*–PAsp with aromatic substitution of the peptide block. In order maximize 3PO entrapment, polymer micelles were prepared by either dialysis or the freeze-drying method. The dialysis method was used to prepare the micelles for block copolymer micelles onto which 3PO was covalently conjugated [PEG–*b*–PHYD/3PO conjugates]. The micelles were characterized by DLS, while drug loading was determined by UV/VIS spectrometry. Comparison in the micelle size indicated no significant differences between the dialysis and freeze-drying methods. Drug loading yields revealed that short lipophilic (aliphatic) groups were more appropriate for increasing 3PO entrapment in the micelles in comparison to hydrophobic (aromatic) pendant groups. In addition, it was shown that 3PO release was faster at pH 5.0 than at pH 7.4.

Micelles containing cis-diaminedichloroplatinum (cisplatin, CDDP) have been synthesized by the self-organization of mPEG–*b*–PLGA polymers in aqueous solutions induced from the complexation of CDDP with the carboxyl groups of the PLGA block. The synthetic approach involved the ROP of the NCA of BLG–NCA initiated by mPEG–NH_2_ [18]. Upon completion of the polymerization, the benzyl groups were removed by dissolving the copolymer in dichloroacetic acid followed by addition of HBr/acetic acid. For the preparation of CDDP-loaded mPEG–*b*–PLG micelles, the copolymer and CDDP were dissolved in distilled water. The size distribution of CDDP-incorporated micelles was evaluated by DLS and TEM. The in vitro release of the CDDP-incorporated micelles was carried out in PBS at pH = 5.5 and pH = 7.4 with 150 mM NaCl. It was found that cisplatin was released from the micelles in a controlled and sustained manner, where faster drug release was observed at pH = 5.5.

More recently, Zheng et al. [19] prepared micellar drug/gene vector nanoparticles from the self-organization of the triblock terpolymer poly(ethylene glycol)–*b*–poly(l-lysine)–*b*–poly(l-leucine) (PEG–*b*–PLL–*b*–PLLeu) with entrapped docetaxel (DTX) and siRNA-Bcl-2. The synthetic procedure first involved the synthesis of the diblock copolymer PEG–*b*–PZLL by the ROP of ZLL–NCA using mPEG–NH_2_ as initiator, followed by the addition of LLeu–NCA using amino-terminated PEG-*b*-PZLL-NH_2_ as a macromolecular initiator. The amphiphilic PEG–*b*–PLL–*b*–PLLeu triblock copolymers were obtained after the deprotection of PEG–*b*–PZLL–*b*–PLLeu by treatment with HBr/HAc in TFA solution. The polypeptide self-assembled into a micellar structure in aqueous solution and exhibited the ability to simultaneous load siRNA and DTX. The micelles loaded with both siRNA as well as DTX showed compact and spherical morphology with a mean diameter of 30–50 nm, as demonstrated by the transmission electronic microscopic (TEM) measurements.

The utilization of polypeptides for the delivery of small interfering RNA (siRNA) provides the possibility to overcome the problems associated with its unstable nature and inefficient cellular uptake. Taking advantage of this development, the Kataoka group [20] presented the synthesis of a triblock polypeptide of the A–B–C type with a shell-forming A-segment composed of hydrophilic poly(ethylene glycol) (PEG), a nucleic acid-loading B-segment containing cationic polylysine (PLL) and a stable core-forming poly{*N*-[*N*-(2-aminoethyl)-2-aminoethyl]aspartamide} (PAsp(DET)) C-segment with the hydrophobic dimethoxy nitrobenzyl ester moiety (DN) in the side chain. This polymer can form micelles that can consolidate the negatively charged siRNA in the PLL segment. To synthesize the polypeptide, PEG–NH_2_ was used as macroinitiator to polymerize *ε*-trifluoroacetyl-l-lysine NCA first, followed by addition of the appropriate amount of BLA–NCA. Furthermore, the aminolysis of the protective groups of PBLA block with diethylenetriamine (DET) occurred, followed by reaction with 4,5-dimethoxy-2-nitrobenzylchloroformate in dichloromethane and deprotection of the ε-trifluoroacetyl group of the polylysine block by addition of NaOH in methanol to provide PEG–*b*–PLL–*b*–PAsp(DET–DN). In this polymer, the DET groups fully reacted with DN. In order to elucidate the role of the hydrophobic C-segment on micellar stability, triblock terpolymers of the type PEG–*b*–PLL(DN)–*b*–PAsp(DET–DN) as well as diblock copolymers PLL–*b*–PAsp(DET-DN) were also synthesized. In the case of the PEG–*b*–PLL(DN)–*b*–PAsp(DET–DN) terpolymer, PLL as well as PAsp(DET) block was partially reacted with the DN groups. siRNA loading was performed by incubation with the preformed polymeric micelles at certain amine/phosphate ratios which was confirmed by gel electrophoresis. It was found that complete complexation of siRNA with the polymer occurred at amine/phosphate ratio = 3. DLS and TEM revealed that the micelles loaded with siRNA were smaller than the blank micelles, which can be attributed to the shrinkage of the PLL layer due to compromised electrostatic repulsion compared to the dimensions of the blank micelles. It was found that the size and ζ-potential of PEG–*b*–PLL–*b*–PAsp(DET–DN) micelles were larger and lower, respectively, than those of PLL–*b*–PAsp(DET–DN) micelles, consistent with the presence of the nonionic PEG shell in the PEG–*b*–PLL–*b*–PAsp(DET–DN) micelles. On the other hand, the partially and randomly hydrophobized triblock copolymer, PEG–*b*–PLL(DN)–*b*–PAsp(DET–DN), did not form such self-assemblies, as evidenced by a substantially lower scattered light intensity compared to PEG–*b*–PLL–*b*–PAsp(DET–DN) and PLL–*b*–PAsp(DET–DN). Thus, it is indicated that a certain amount of sequential hydrophobic moieties in the C-segment may be crucial for stable micellar formation.

Lv et al. [21] reported the synthesis of poly(ethylene glycol)–*b*–poly(l-glutamic acid-*co*-l-phenylalanine) (mPEG–*b*–P(LGA–*co*–Phe)) and used it as a nanovehicle for DOX. The polymer was synthesized using mPEG-NH_2_ as macroinitiator for the copolymerization of BLG–NCA and Phe–NCA, followed by deprotection of BLG by treating with HBr/acetic acid. The amphiphilic diblock terpolymer formed micelles in aqueous solution. The encapsulation of DOX occurred by dissolving the polymer and the drug in distilled water, followed by removal of free DOX by dialysis. Finally, the solution was freeze-dried to obtain the DOX-loaded nanoparticles (DOX–NP). The PEG block formed the shell of the nanoparticles in order to ensure prolonged circulation in the blood compartment. The poly(phenylalanine) domain was used for stabilizing the nanoconstruct through hydrophobic/aromatic interactions, while the PLGA block was used to provide electrostatic interactions with the cationic drug. The copolymer self-assembled into micellar-type nanoparticles, and DOX was successfully loaded by mixing DOX∙HCl and the copolymer in the aqueous phase. The stability of the nanoparticles was a key factor for the successful DDS. Increasing the overall hydrophobicity of the block copolymer in the nanoparticles led to a significant increase in the stability of the nanoparticle and its anticancer activity.

**Figure 5:**
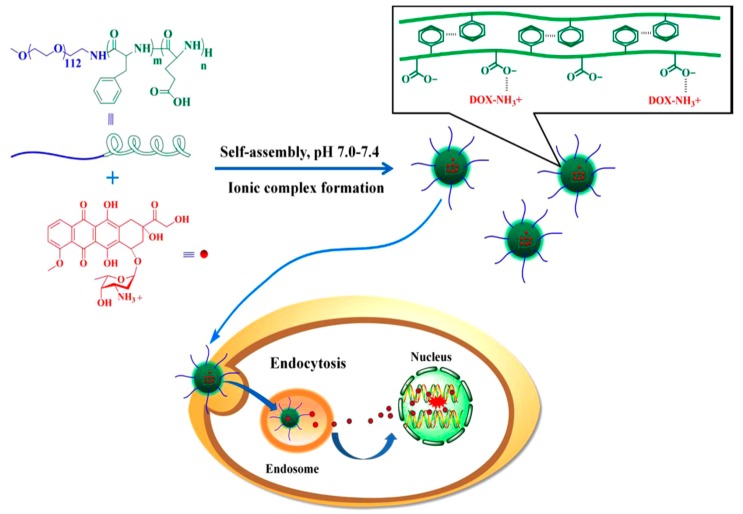
Preparation and interaction mechanism of doxorubicin (DOX)-nanoparticles with cancer cells [21].

DOX release from mPEG–*b*–P(LGA–*co*–Phe) nanoparticles was investigated using a dialysis method. The release was characterized by an initial fast release, followed by much slower release. At pH 7.4, only 22% of total drug was released before the release profile has virtually plateaued. This result suggests that DOX–NP maintained strong drug–polymer electrostatic interactions under physiological conditions. At pH 6.8, 29% of DOX was released after 12 h. However, 60% of DOX was released within 12 h when pH was decreased to 5.5. Finally, total drug release at pH 5.5 was three times higher than that at pH 7.4. This result demonstrates that the release of DOX from mPEG–*b*–P(LGA–*co*–Phe) nanoparticles is pH sensitive. However, the release did not reach 100% even at pH 5.5. Consequently, the study has shown that the self-assembled polymer/drug complexes driven by electrostatic interactions may be a promising drug delivery system for cancer therapy (Figure 5).

In 2014, Kim et al. [22] described the synthesis of cancer–recognizable magnetic resonance imaging (MRI) contrast agents (CR–CAs) with the utilization of pH-responsive polymeric micelles. The ability of CR–CAs to self-assemble into micelles was based on well-defined amphiphilic block copolymers, consisting of a mixture of polymers, i.e., methoxy poly(ethylene glycol)–*b*–poly(l-histidine) (PEG–*b*–PHis) and methoxy poly(ethylene glycol)–*b*–poly(l-lactic acid)-diethylenetriaminopentaacetic acid-gadolinium chelate (PEG–*b*–P(l-LA)–DTPA–Gd). The synthesis of PEG–*b*–P(l–LA) diblock copolymer occurred by ROP of l-lactide with PEG–OH as initiator and stannous octate as catalyst, followed by reaction with DTPA dianhydride and chelation of Gd to afford PEG–*b*–P(l–LA) –DTPA by addition of GdCl_3_·6H_2_O. The PEG–*b*–Phis copolymers were synthesized by the ROP of dinitrophenyl-protected histidine NCA that was polymerized with mPEG–NH_2_ as the macroinitiator, followed by deprotection with mercaptoethanol. The polymers were characterized by ^1^H NMR spectrometry and FT-IR, while the content of Gd in the samples was estimated by inductively coupled plasma–optical emission spectrometry (ICP–OES) analysis.

The pH-sensitive CR–CAs were prepared by mixing PEG*–b–*P(l-LA)–DTPA–Gd:PEG-P(l-His) in a 1:4 *w*/*w* ratio. These NPs were compared to pH-insensitive micelles resulting from the self-organization of PEG–P(l-LA)–DTPA–Gd polymers IIns–Cas). The CR–CAs and Ins–CAs were analyzed by dynamic light scattering (DLS) and TEM. It was shown that the pH-responsive polymeric micelles enable more accurate and rapid cancer diagnosis, as compared to those without pH sensitivity. This observation was explained as follows: when CR–CAs reach the acidic tumoral environment, they are deconstructed in the acidic tumoral environment (pH 6.5) due to the protonation of the imidazole groups of PHIS blocks. At neutral pH (pH 7.4) the CR–CAs could behave as stable micelles with decreased sensitivity, whereas at pH 6.5, the CR–CAs break apart into charged water-soluble polymers with increased sensitivity due to the interaction between water molecules around the acidic tumor tissue, leading to exposed Gd^3+^ from the micelle core. The images revealed that the CR–CAs had a spherical shape with rather small size, equal to 40 nm at pH 7.4, which is important for easy penetration of the tumor tissue, however their size decreased to < 5 nm at pH 6.5. In contrast, Ins-Cas did not exhibit any changes in size at various pH values.

More recently, Jia et al. [23] described the synthesis of novel, degradable and pH-responsive 4-arm star copolymer featuring a porphyrin core, where each arm is an amphiphilic poly(ε-caprolactone)–*b*–poly(l-lysine) (SPPCL–*b*–PLL) copolymer. The synthetic approach of the copolymers involved two steps. In the first, 4-arm star-shaped PCL with porphyrin core (SPPCL) was prepared with the use of tetrahydroxyethyl-terminated porphyrin as initiator for the ROP of CL. The polymerization occurred using a catalytic amount of Sn(Oct)_2_. The terminal hydroxyl groups of SPPCl were then converted to bromine with the use of 2-bromoisobutyryl bromide, followed by reaction with sodium azide NaN_3_ to obtain SPPCLN_3_. Finally, the SPPCLNH_2_ was obtained through the reduction of azide group using triphenylphosphine.

In the second step, the polymerization of ZLL–NCA was initiated by the tetrafunctional macroinitiator SPPCLNH_2_, followed by deprotection of the peptide blocks by reaction with trifluoroacetic acid. These copolymers could self-assemble into micelles, with the hydrophobic porphyrin centered PCL arms as the inner core and the hydrophilic PLL blocks as the outer corona. Transmission electron microscopy (TEM), atomic force microscopy (AFM) and dynamic light scattering (DLS) were used to determine the molecular characteristics of the micelles.

Copolymers with shorter hydrophilic chains revealed a uniform spherical formation, whereas their longer hydrophilic counterparts were less uniform. The pH-responsiveness of the micelles is due to the protonation of PLL block. At pH values lower than the pKa of PLL (pKa = 10.6), the positively charged amine groups enhanced the interaction with water molecules, thereby increasing the solubility and the stability of the core–shell micelles. Increasing the pH above 10.6, the random coil formation of the PLL converted into *α*-helix, decreasing their solubility and therefore their stability. Due to the optical properties of the porphyrin core, the micelles revealed a certain degree of photodynamic therapy (PDT) effects on tumor cells, indicating that these micelles are a very promising application as drug delivery system for cancer treatment.

**Figure 6:**
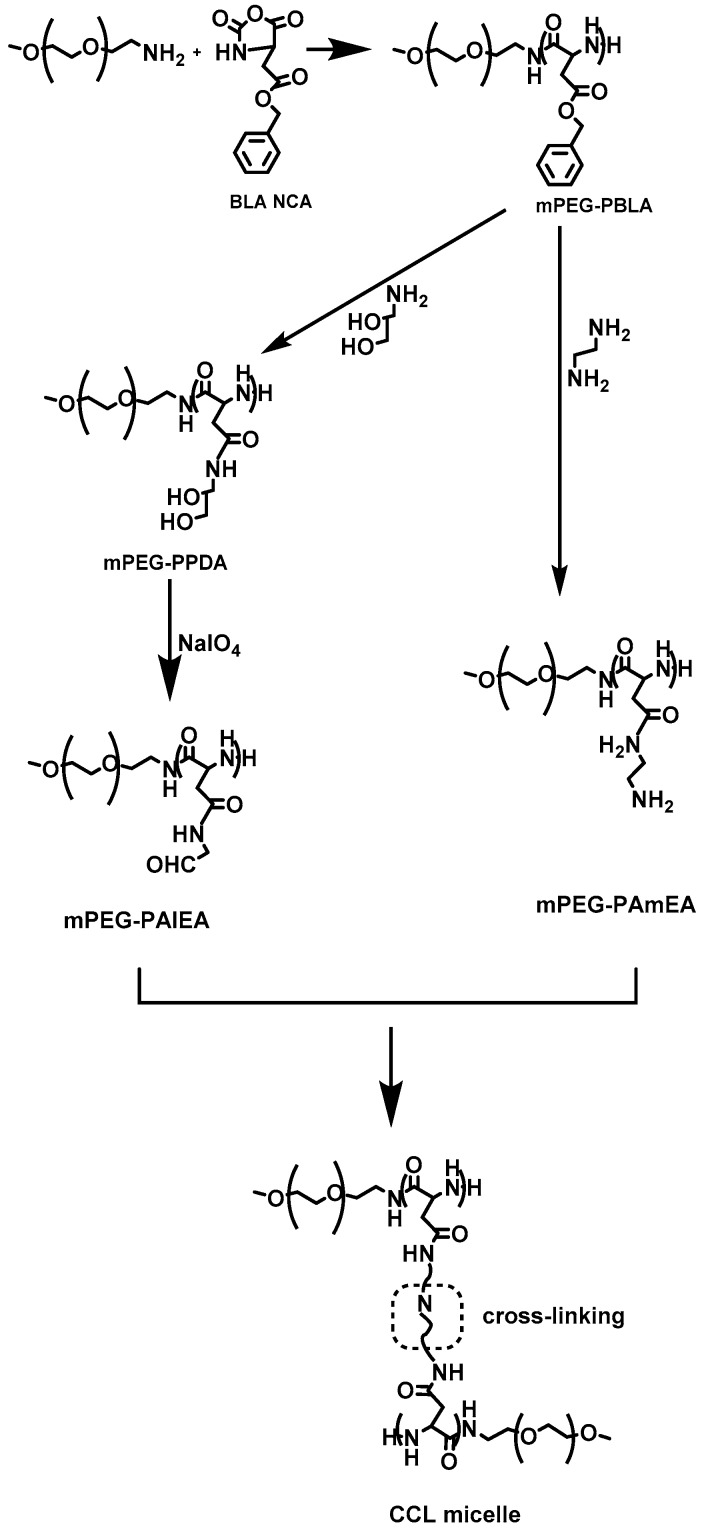
Synthesis of mPEG–PAmEA, mPEG–PAlEA and core-crosslinked mPEG–PA micelles.

In 2014, Wang et al. [24] reported the synthesis of a variety of amphiphilic methoxy-poly(ethylene glycol)–*b*–poly(aspartamide)s (mPEG–*b*–PAsp) copolymers, with various amide substitution groups, the copolymers were used for the formation of pH-sensitive micelles for controlled DOX delivery. The synthesis of the copolymers first involved the preparation of *α*-methoxy, ω-amino–poly(ethylene glycol) (mPEG–NH_2_), by the transformation of the terminal hydroxyl group of mPEG–OH into mPEG–NH_2_ that was used as the macroinitiator for the ROP of BLA–NCA, to afford mPEG–*b*–PBLA. Then, aminolysis occurred on the polypeptidic block after treatment with 1,2-ethanediamine to give methoxy-poly(ethylene glycol)–*b*–poly(ethaneamine l-aspartamide) (mPEG–*b*–PAsp(EN)). Methoxy-poly(ethylene glycol)–*b*–poly(propanediol l-aspartamide) (mPEG–*b*–PAsp(PD)) was successfully synthesized by aminolysis with 3-amino-1,2-propanediol of mPEG–*b*–PBLA. Finally, methoxy-poly(ethylene glycol)–*b*–poly(aldehyde ethyl aspartamide) (mPEG–*b*–PAsp(AlEA)) was synthesized by treating mPEG–*b*–PAsp(PD) with NaIO_4_ (Figure 6). DOX-loaded core-cross-linked micelles were prepared by mixing mPEG–*b*–PAsp(AlEA) with mPEG–*b*–PAsp(PD) in DMSO in the presence of DOX. The crosslinked core resulted after reaction of the amine groups of mPEG–*b*–PAsp(PD) with the aldehyde groups of mPEG–*b*–PAsp(AlEA) to form pH-labile imine bonds. The core-cross linked micelles were found to be stable at pH 7.4, while at lower pH the imine bonds began to be cleaved, resulting in the quick release of DOX.

The same year, Yang et al. [25] reported the synthesis of 3-miktoarm star copolymers of the Y-shaped methoxy–poly(ethylene glycol))_2_–poly(l-glutamic acid ((mPEG)_2_–PLGA) and its precursor methoxy–poly(ethylene glycol))_2_–poly(*γ*-benzyl-l-glutamate ((mPEG)_2_–PBLG). The synthetic approach involved the synthesis of the macroinitiator (mPEG_5k_)_2_–NH_2_, which involved reaction of mPEG_5k_–OH with hexamethylenediisocyanate (HDI) to form mPEG_5k_–NCO, followed by coupling of two mPEG_5k_–NCO with *N*-Boc-serinol (NBS) to obtain mPEG_5k_–NHBoc. Finally, the (mPEG_5k_)_2_–NH_2_ macroinitiator featuring an in-chain amine group resulted after deprotection of the Boc group by treatment with HBr/HAc/CF_3_COOH. Three copolymers with different PBLG molecular weight subsequently synthesized (mPEG)_2_-PBLG_14, 20, 35_ via the ROP BLG–NCA initiated by the in-chain amine functionalized (mPEG_5k_)_2_–NH_2_ macroinitiator. Finally, the benzyl groups on the PBLG block were removed by HBr/ HAc/ CF_3_COOH to afford the corresponding 3-miktoarm star copolymer (mPEG)_2_–PLGA.

In order to compare the properties of the miktoarm stars, the authors also synthesized the linear analogues of the type (mPEG)–*b*–PLGA with molecular weight of mPEG equal to the one of a single arm of the miktoarm star. Characterization results by SEC confirmed the successful synthesis of the linear polymer and the stars, showing that they exhibited a high degree of molecular and compositional homogeneity. Both conjugates could self-assemble into micelles and revealed an enhanced anti-cancer efficacy. (mPEG)_2_–PBLG could self-assemble into micelles in aqueous solution with the hydrophilic mPEG as the outer shell and the hydrophobic PBLG as the inner core of the micelle. Both Y-shaped and linear mPEG–*b*–PBLG self-assembled into micelles, which exhibited a spherical morphology as shown by TEM. In addition, DLS analysis was used to determine the sizes of the (mPEG)_2_–PBLG_14, 20, 35_ which were 123, 117, 99 nm respectively, higher than that of their linear counterparts.

Paclitaxel (PTX) was chemically conjugated with (mPEG)_2_–PLGA to give (mPEG)_2_–PLGA–PTX by esterification of the carboxyl groups of PLGA with the hydroxyl groups in the 2’ position of PTX, in the presence of DCC and DMAP. The PTX content was about 20% (*w/w*), and the PTX coupling efficiency was calculated by ^1^H NMR analysis at 90% (*w/w*). Similarly, the (mPEG)_2_–PLGA–Pt conjugate was prepared by the coordination of the carboxyl groups in the PLGA segment and the central Pt atom of cisplatin. ICP-MS analysis indicated a 15% (*w/w*) Pt content in the conjugate. Both (mPEG)_2_–PLGA–PTX and (mPEG)_2_–PLGA–Pt self-assembled into micelles with the hydrophilic mPEG as the outer shell and the hydrophobic PLGA–PTX or PLGA–Pt as the core of the micelles. The spherical shape of the micelles was confirmed by TEM analysis, and it was found that the average particle size which was about 50 nm for (mPEG)_2_–PLGA–PTX and about 45 nm for (mPEG)_2_–PLGA–Pt. The drug release occurs at low pH inside the cells. In vitro studies against MCF-7, HeLa, and SMMC cell lines showed enhanced antitumor activity, while in vivo antitumor activity of (mPEG)_2_–PLGA–Pt showed excellent antitumor efficacy against H22 cancer cells.

In 2014, Gyu Ha Hwang et al. [26] reported the synthesis of pH-responsive core–shell polymer micelles through the self-assembly of poly(ethylene glycol)(PEG)–*b*–poly(l-3,4-dihydroxyphenylalanine) (PEG–*b*–PDOPA). The amphiphilic block copolymer was synthesized by the ROP of di-*O*,*O*’-acetyl-l-DOPA-*N*-carboxyanhydride ((AC2)–DOPA–NCA) with CH_3_O–PEG–NH_2_ as the macroinitiator, followed by deprotection of the hydroxyl groups. The polymer was characterized by size exclusion chromatography as well as ^1^H NMR spectroscopy. The polymer forms core–shell micelles, with the hydrophobic block PDOPA in the core. The catechol groups of hydrophobic PDOPA crosslink in the presence of Fe^3+^, depending on the pH. At pH = 7.4, the Fe^3+^ forms bis-catechol–Fe^3+^ core cross-links. DTX was loaded to core-cross-linked polymer micelles (DTX–CLPMs) through dialysis. Upon endocytosis, coordination of catechol–Fe^3+^ transforms from the bis-complex to the mono-complex in response to endosomal pH (pH 5.0). At low pH, the crosslinks break and trigger the facilitated intracellular drug release. The authors also studied the stability of DTX–CLPM_S_ and DTX-loaded non-crosslinked (DTX–NPM_S_) in fetal bovine serum (FBS) using DLS. It was found that catechol-Fe^3+^ core cross-linked could improve the structural stability of polymer micelles under serum conditions. Therefore, the crosslinks in the DTX-loaded PDOPA cores were found not only to enhance micellar stability against micelle-destabilizing conditions but also to ensure efficient drug release in extracellular environments (pH 7.4).

A graft copolymer that can fold into nanostructures in a protein-like manner was synthesized in two steps by Tai et al. [27]. In the first step, the backbone of the graft copolymer, a multifunctional polymer based on PEG bearing amine groups, was synthesized. The synthesis involved the polymerization of the “tee-joint”-like monomer and bifunctional NHS–PEG–NHS, through the polycondensation reaction between the two NHS groups of PEG and the two amine groups of (*N,N*-bis(2-aminoethyl)-*N*-[2-(tert-butylcarboamoyl)ethyl-amine)] The BOC protecting group of (*N,N*-bis (2-aminoethyl)-*N*-[2-(tert-butylcarboamoyl)ethyl-amine)] was then cleaved by TFA, and the amine groups were activated with N-trimethylsilyl (TMS) by reacting with *N,O*-bis(trimethylsilyl) acetamide and were used as the initiator for the graft polymerization of *g*-camptothecin-glutamate *N*-carboxyanhydride (Glu(CPT)–NCA). The nanoprecipitation methodology was used in order to encapsulate DOX. The structure of the micellar nanocarriers was characterized by TEM and the size by DLS. Furthermore, the core shell structure of the nanocarriers was characterized by ^1^H NMR.

It was found that less than 10% of CPT was released from nanocarriers, as monitored in 100% mouse serum by high performance liquid chromatography. The release rate of camptothecin from the nanoparticle and Glu(CPT)–OH was compared. It was found that less than 10% of CPT was released from the nanocarriers in the presence of the mouse serum within 6 days, indicating its high serum stability. In contrast, Glu(CPT)–OH showed a burst CPT release profile under the same conditions, indicating that the presence of the PEG shell of the nanoparticle was able to effectively shield off esterase attack and prevent premature drug release. The authors also showed that the encapsulated DOX displayed preferential release under weak-acid conditions. This was attributed to the re-protonation of the DOX amino group and its subsequently higher aqueous solubility at a lower pHs, indicating that the drugs would be released within the endosome upon cell internalization. In vivo studies showed that the graft copolymer folded nanocarrier produced measurable inhibitory effect on tumor growth, and that their therapeutic efficacy might be attributed to the superior accumulation at tumor sites and to insignificant in vivo toxicity as well.

In order to overcome the barriers that reduce the effectiveness of the combination of PTX and CDDP in many types of cancer and to distinguish the side-effects during cancer treatment, Song et al. [28] proposed the synthesis of a triblock copolymer poly(ethylene glycol)–*b*–(poly(l-glutamic acid) –*b*–poly(l-phenylalanine) (mPEG–*b*–PLGA)–*b*–PPhe) that forms a micelle for the co-delivery of both drugs. PTX was entrapped in the hydrophobic core of PPhe, and CDDP chelated due to its hydrophilic metal complex nature to the PLGA middle shell, while mPEG formed the outer corona. *α*-Methoxy-*ω*-amino-poly(ethylene glycol) was the macroinitiator for the ROP through the sequential addition methodology of the monomers BLG–NCA and Phe-NCA, followed by deprotection of the benzyl groups of PBLG by treatment with HBr in acetic acid. For the preparation of the micelles and loading of the drugs, the polymer and PTX were both dissolved in DMSO followed by addition of phosphate buffer. The solution was then dialyzed against distilled water followed by centrifugation. The loaded micelles remaining in the supernatant solution were filtered through a 0.45 um filter and freeze-dried. The resulting PTX loaded powders were dissolved in distilled water and CDDP was added at pH = 8. The mixture was left to react at 37 °C followed by dialysis to afford the dual drug loaded micelles.

Based on this strategy, four kinds of drug-loaded micelles were produced: micelles loaded only with PTX, micelles loaded only with CDDP, and micelles loaded with both drugs at two loading contents of PTX/CDDP 0.3 and 0.15 (*w/w*). All 4 types had hydrodynamic radii between 80 and 100 nm. It was also found that after CDDP loading, the size was decreased and zeta potential became less negative due to cross-linking induced by the chelation of Pt^II^ in the middle shell of PLGA. Furthermore, in vitro release experiments showed that the CDDP release was faster at pH 5.5 due to the protonation of the carboxylic groups of PLGA, which prevented Pt chelation. The PTX release was faster than that of CDDP in the same conditions for the micelles loaded with one drug only, while the presence of Pt^II^ crosslinks in the micelles loaded with both drugs resulted in the deceleration of PTX release.

In 2016, Rao et al. presented the synthesis of individual and mixed paclitaxel/rapamycin (PTX/RAP) micelles [29]. These micelles were formed with PTX and/or RAP and poly(ethylene glycol)-*b*-poly(*β*-benzyl-l-aspartate) copolymer via a pH-sensitive linker. The BLA–NCA was synthesized according to Fuchs-Farthing method. The synthetic approach for the copolymers involved the ROP of BLA–NCA initiated by the terminal primary amine group of *α*-aminopropyl-*ω*-methoxy-poly(ethylene glycol). The resulting copolymer PEG–*b*–PBLA was reacted with hydrazine to afford the PEG–*b*–P(Asp-Hyd) (PEG–*b*–HYD) through an ester-amide exchange reaction. In a following step, the hydrazine groups were reacted with the ketone group of levulinic acid (LEV). LEV is an aliphatic linker which was used as a spacer group on PEG–HYD to conjugate the PTX and RAP drugs in the polymer. Finally, PTX or RAP were conjugated on PEG–*b*–LEV via the formation of an ester bond between the carboxyl group of the embedded LEV and a hydroxyl group of the drugs to provide PEG–*b*–P(Asp–Hyd–LEV–PTX) or PEG–*b*–P(Asp–Hyd–LEV–RAP).

**Figure 7:**
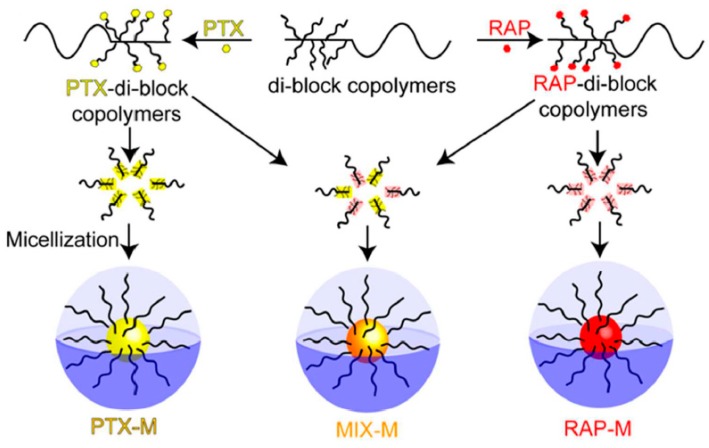
Paclitaxel (PTX) micelles, Rapamycin micelles and mixed micelles conjugated to diblock copolymer poly(ethylene glycol)–*b*–poly(*β*-benzyl-l-aspartate) [29].

The polymers were characterized by ^1^H NMR and SEC analysis, which confirmed the successful synthesis. The characterization results showed that PEG-*b*-P(Asp–Hyd–LEV–PTX) had a 37.5% degree of drug substitution, while PEG–*b*–P(Asp–Hyd–LEV–RAP) exhibited 16.40% substitution. In addition, reversed-phase high pressure liquid chromatography (RP-HPLC) analysis was used to confirm the absence of unreacted PTX and RAP. By using SEC analysis featuring a triple detection system, the hydrodynamic radius (Rh), the intrinsic viscosity [η] as well as the Mark-Houwink`s constant (a) were obtained in aqueous solutions. The measurements revealed that the [η] of the three polymers PEG–p(Asp–Hyd–LEV), PEG–p(Asp–Hyd–LEV–PTX), PEG–p(Asp–Hyd–LEV–RAP) had different values, indicating a heterogeneity not only in the composition, but also in the interaction with the solvent. PTX micelles (PTX–M), RAP micelles (RAP–M) and PTX:RAP 1:1 molar ratio mixed micelles (MIX–M) were prepared and characterized by DLS and TEM. The size of PTX–M, RAP–M and MIX–M were 41.9, 90.1 and 102.3 nm, respectively (Figure 7).

In addition, the release profile of the drugs from the micelles was studied at pH 7.4 and 5.5, where it was shown that release was higher at pH 5.5. This was attributed to the quick cleavage of LEV from the polymer backbone at acidic pH. Therefore, the micellar systems could be used as drug nanocarriers for the tandem delivery of PTX and RAP in both tumor microenvironments as well as inside ovarian cancer cells. In vitro and in vivo studies showed that the conjugated PTX, RAP or MIX–M micelles led to the treatment of ovarian cancer through both cytotoxic effects to cancer cells and simultaneous angiogenesis inhibition.

More recently, Suo et al. [30] reported the three-step synthesis of the amphiphilic copolymer poly(l-phenylalanine)–*b*–poly(l-lysine-*g*-methoxypolyethylene glycol)–*b*–poly(l-phenylalanine) (PPhe–*b*–(PLL-*g*-mPEG)–*b*–PPhe). In the first step, *α*-methoxy ω-aldehyde functionalized poly(ethylene glycol) (mPEG–CHO) was synthesized by the oxidation of mPEG–OH with dimethyl sulfoxide(DMSO)/acetic anhydride. PZLL was subsequently synthesized by the ROP of ZLL–NCA using ethylenediamine as a difunctional initiator, followed by addition of Phe–NCA after the completion of the polymerization of the first monomer, to afford PPhe–*b*–PZLL–*b*–PPhe. Deprotection of PZLL then took place after cleavage of the benzyloxycarbonyl protecting groups and treatment with trifluoroacetic and hydrobromic acid-acetic acid (HBr–HAc). Finally, PPhe–*b*–(PLL–*g*–mPEG)–*b*–PPhe was prepared by reductive amination between the amine groups of PLL and the aldehyde groups of mPEG–CHO. The terpolymers were molecularly characterized by ^1^H NMR.

In addition, blank and DOX-loaded PPhe–*b*–(PLL–*g*–mPEG)–*b*–PPhe nanomicelles were prepared by the dialysis method. The ability of DOX-loaded nanomicelles to condense P-gp siRNA was studied. To investigate the amount of the condensed P-gp siRNA on the micelles, the authors employed an agarose gel electrophoresis assay, using blank mPEG–*g*–PLL–*b*–PPhe nanomicelles as a control. The blank and DOX-loaded nanomicelles started to form complexes with P-gp siRNA molecules at low *N*/*P* ratios, and complete complexation of P-gp siRNA molecules occurred at the *N*/*P* ratios between 2 and 4. The reduced complexation of DOX-loaded nanomicelles with P-gp siRNA resulted from the reduced positive charge density of nanomicelles caused by DOX loading. The loading of DOX and siRNA increased nanomicelle volume.

The DOX and siRNA release profiles showed an obvious initial burst release followed by a sustained release. It was also noted that the release changed significantly with pH. Enhanced DOX and P-gp siRNA release from the nanomicelles could be clearly observed at pH 5.0 compared to that at pH 7.4. At pH 7.4, approximately 32.5% of the DOX loaded in nanomicelles was released after 42 h, while 34.0% of the P-gp siRNA loaded was released at the same time. The percentages of DOX and siRNA released at pH 5.0 over the same time were 48.6% and 64.5%, respectively. The results indicate the synergistic therapeutic effects of the terpolymer as an effective nanocarrier for the co-delivery of hydrophobic chemotherapeutic drugs and genetic material.

Ramasamy et al. [31] reported a method for co-loading of DOX and quercetin (QUR) using a polypeptide nanoconstruct of the poly(phenylalanine)–*b*–poly(l-histidine)–*b*–poly(ethylene glycol) type. The smart pH-sensitive nanovehicle was used for synergistic endolysmosal delivery of DOX/QUR in cancer chemotherapy. The authors used an mPEG_113_–NH_2_ as macroinitiator for the sequential two-step ROP of Bz–His–NCA initially followed by Phe–NCA. Deprotection of histidine residues was achieved with metallic sodium. SEC as well as ^1^H NMR analysis confirmed the synthesis of the triblock. For the preparation of DOX/QUR loaded micelles, DOX hydrochloride was initially converted to DOX with the addition of triethylamine and was dissolved in DMSO with the addition of QUR. The triblock polypeptide was dissolved also in DMSO and was then mixed with the dissolved drugs. Dialysis and freeze-drying were carried out to provide the loaded micelles. The entrapment efficiency for both drugs was more than 90%, and the active loading capacity of 9.5% ± 1.25% *w/w* was achieved for QUR and 8.6% ± 1.39% *w/w* for DOX. DLS and Zeta-potential revealed increase in size and decrease in charges upon pH decrease, while the drug release was faster at pH 5.5 than at 7.4. The slight increase in particle size for the loaded micelles can be attributed to the incorporation of the drugs in the hydrophobic core. Deformation of the inner core/corona of the loaded micelles was induced by pH alteration. It was shown that the nanoparticles loaded with combined drugs presented better antitumor activity across all cancer cells in all concentrations tested compared to each single drug. Enhancement of the cytotoxic potential of DOX by QUR was achieved since marked cell apoptosis, change in cell cycle patterns and inhabitation of the migratory capacity of sensitive and resistant cancer cells were observed in the representative in vivo and in vitro experiments.

## 4. Temperature Responsive Micelles

**Figure 8:**
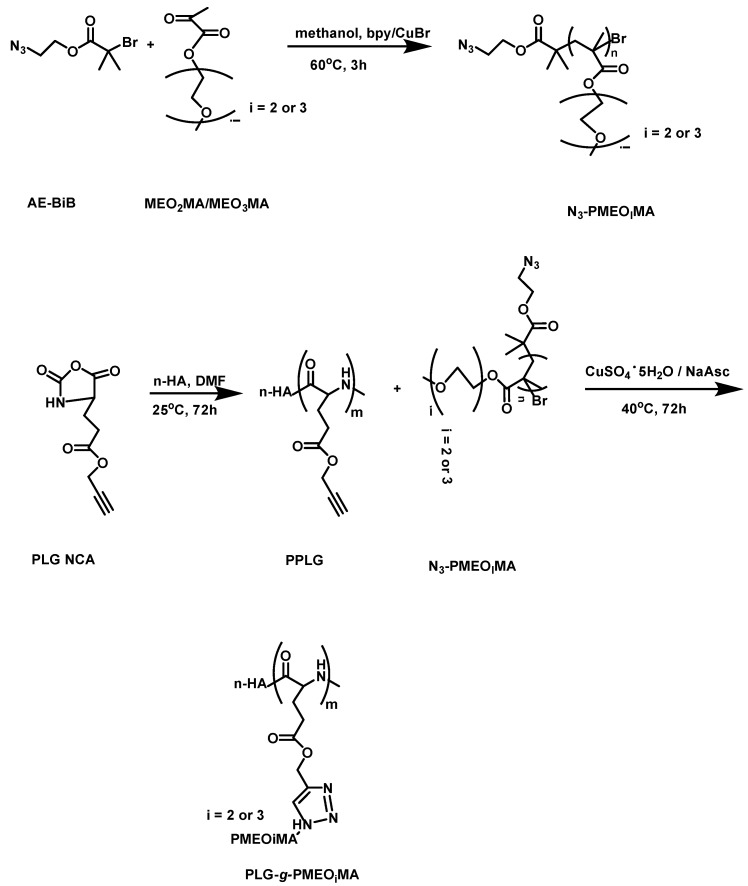
Synthetic route to PLG-*g*-PMEOiMA.

In 2013, Ding et al. [32] reported the synthesis of a series of thermo-responsive “hairy-rod” polypeptides that form micelles and are capable of the delivery of anti-tumor drugs. Their synthesis involved a combination of different strategies. Initially, *γ*-propargyl-l-glutamate (PLG–NCA) was used in order to synthesize poly(*γ*-propargyl–l-glutamate) (PPLG) as the polypeptide backbone, with *n*-hexylamine as the initiator. In the next step, a series of azide-terminated polymers of 2-(2-methoxyethoxy)ethyl methacrylate and 2-(2-(2-methoxyethoxy)ethoxy)ethyl methacrylate (N_3_PMEO_i_MA) was performed through ATRP. The initiator 2-azidoethyl bromoisobutyrate was synthesized, using 2-azidoethanol in the presence of triethylamine, by the dropwise addition of 2-bromoisobutyryl bromide in THF. Then, the initiator 2-azidoethyl bromoisobutyrate was mixed with bpy, MEO_2_MA and MEO_3_MA in the presence of CuBr at 60 °C to give the corresponding polymers bearing a terminal azide group. Finally, MEO_2_MA and MEO_3_MA were “grafted onto” PPLG in the presence of copper through click chemistry (Figure 8). FT-IR, ^1^H NMR and SEC were used to confirm the successful synthesis. The DP of PLG was constantly 40, and the DP of MEO_2_MA/MEO_3_MA was 27/0, 18/9, 13/13, 7/18, 0/26. The thermo-responsive properties as well as the secondary structure of the polymers were dependent on the MEO_2_MA:MEO_3_MA ratio in the polymer side chains. Increased MEO_3_MA content in the side chain resulted in lower *α*-helix formation for the polymer and an increase in lower critical solution temperature (LCST) and cloud point (CP). The polymers self-assembled into micelles in PBS, with PLG in the core while PMEO_i_MA formed the shell.

**Figure 9:**
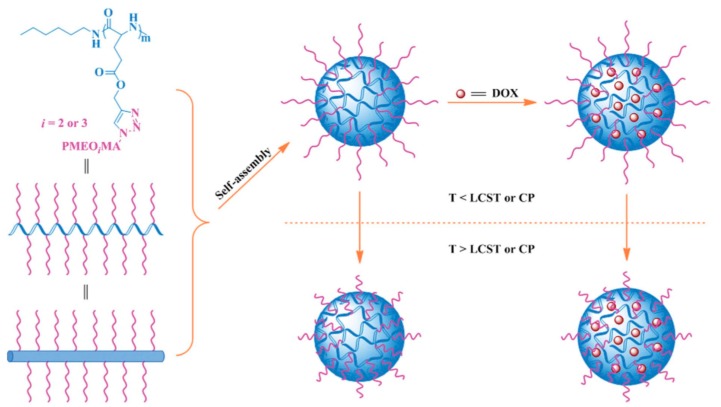
Self-assembly of “hairy-rod” polypeptides, DOX loading and responsiveness to thermal stimuli [32].

For the DOX loading, PLG_40_-*g*-P(MEO_2_MA_18_–*co*–MEO_3_MA_9_) and PLG_40_–*g*–P(MEO_2_MA_7_–*co*–MEO_3_MA_18_) were selected, with DLC and DLE equal to 40.4 and 15.8 wt % respectively. DOX was encapsulated using the nanoprecipitation methodology in the presence of triethylamine. It was shown that the lower hydrophilicity of PMEO_2_MA compared to PMEO_3_MA resulted in the higher DLC and DLE. In PBS at pH 7.4, the release rate of the drug was higher at 20 than 37 °C, because of the collapsed conformation at temperatures above LCST and CP. The increase of MEO_3_MA content in the polymer’s side chain resulted in increased drug release. In addition, the release rate of the drug was higher at lower pH (Figure 9).

Johnson et al. [33] presented the synthesis of a series of novel temperature-responsive polymers of the poly(*N*-isopropylacrylamide)–*b*–poly(l-histidine) [p(NIPAM)–*b*–p(His)] type with 55 monomeric units of p(NIPAM) and 50, 75, 100 and 125 monomeric units of p(His). The authors employed a combination of the polymerization strategies of reversible addition-fragmentation chain transfer polymerization (RAFT) and ROP and envisioned the perspective of the use of this material as a pH- and temperature-responsive antitumor drug carrier. Initially, tosyl-protected 2-amino ethanol was synthesized, followed by coupling with 1-dodecanethiol in the presence of DMAP and DCC, resulting in the tosyl-protected RAFT chain transfer agent 2 (CTA-2). NIPAM, AIBN and CTA-2 were used for the preparation of *ω*-tosylated pNIPAM. The polymer was dissolved in methanol followed by addition of HBr in acetic acid for the deprotection of the polymer, followed by removal of the HBr after the pH adjustment of its solution in methanol at 11, resulting in pNIPAM–NH_2_. This was used as the macroimitiator for the polymerization of *N*-*im*-benzyl-l-histidine NCA (Bn–His–NCA), followed by deprotection of the benzyl groups of His by treatment with trifluoroacetic acid and a 33 wt % solution of HBr in acetic acid. For micelle production, the polymer was dissolved in DMAc, followed by the addition of deionized water, dialysis and lyophilization.

The encapsulation of DOX was performed by the nanoprecipitation methodology, by dissolving DOX in DMAc with triethylamine, addition to the polymer solution also in DMAc and addition of water, followed by dialysis to remove DMAc as well as the unencapsulated DOX. They found that p(NIPAM) block of the copolymers has a reversible thermoresponse near its LCST because of its aggregation and the coil to globule transition, which reveals that the behavior of the polymers is analogous of the p(NIPAM) homopolymer. It is worth noticing that by increasing the histidine block, the LCST increased constantly. Temperature and pH responsiveness was also ascertained by the DLS measurements. As the solution temperature increased to the corresponding LCST of each polymer and as the solution pH decreased, the aggregate size increased. The pH responsiveness is related to the histidine block, which is a pH-dependent material. All the polymers formed spherical micelles, an observation which was irrelevant to the pH. The size of those micelles was bigger when DOX was encapsulated. As more histidine units were added to the polymer, the drug-loading capacity of the micelles increased because p(His) was located at the core of the micelle. At 37 °C, the DOX release rate was higher in comparison with that at 15 and 25 °C due to the higher hydrophobicity of the p(NIPAM), resulting in lower solubility of the polymer in water and destabilization of the micelle. The release rate was also higher at more acidic conditions, because the imidazole rings of the histidine block were protonated at lower pH and the hydrophobic interactions were weaker.

## 5. Redox-Responsive Micelles

**Figure 10:**
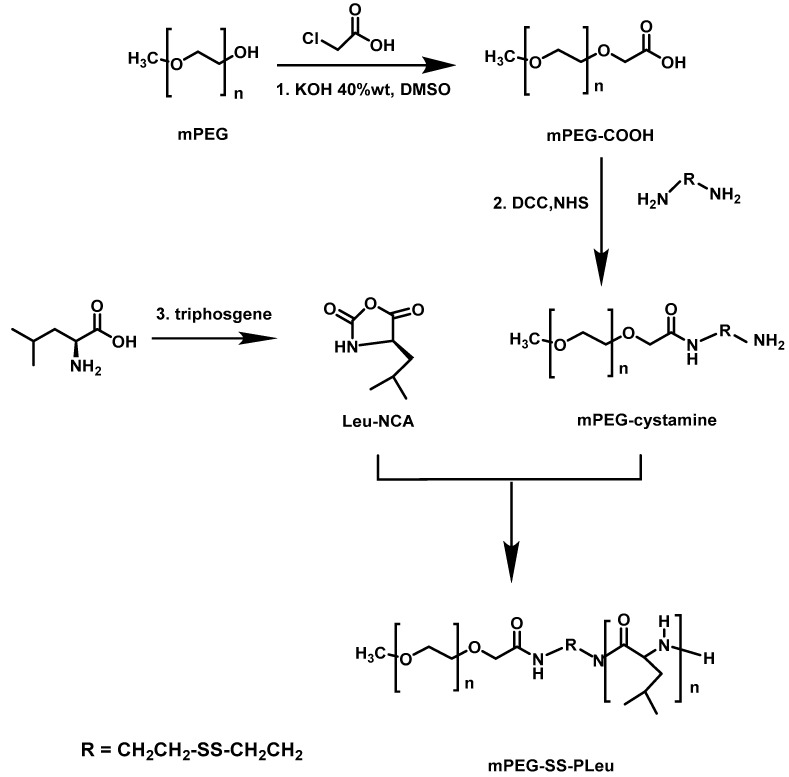
Synthesis of the mPEG–SS-Pleu copolymer via disulfide bond conjugation.

A novel reduction-sensitive sheddable micelle based on disulfide-linked hybrid methoxy-poly(ethylene oxide)–SS–*b*–polyleucine (mPEG–SS–*b*–PLLeu), was utilized for intercellular drug delivery [34]. The synthetic approach of the polymers involved the preparation of the macroinitiator mPEG-cystamine, followed by the ROP of LLeu–NCA. The macroinitiator was synthesized by reaction of mPEG–OH with chloroacetic acid to give mPEG–COOH, followed by reaction with cystamine in the presence of DCC and NHS (Figure 10). The polymers were characterized by SEC, FT-IR showing the successful synthesis. DLS, TEM, ^1^H NMR and fluorescence techniques were also used in order to confirm the micelle formation of the polymers in aqueous solutions. DOX was encapsulated through the nanoprecipitation methodology. The release profiles of DOX from the micelles showed that the drug release rate was much faster in the presence of DTT than in its absence, due to disulfide bond cleavage.

In 2012, Ding et al. [35] reported the two-step synthesis of disulfide-linked block copolymers of methoxy poly(ethylene glycol)–SS–poly(3-benzyloxycarbonyl-l-lysine) (mPEG–SS–*b*–PZLL) in order to develop biocompatible reduction-responsive micellar systems for efficient intracellular drug delivery. The macroinitiator mPEG–SS–NH_2_ was synthesized in the first step through a procedure similar to that presented by Ren et al. [10]. mPEG–OH was reacted with succinic anhydride to afford mPEG–COOH, followed by reaction of cysteamine in the presence of 1-ethyl-3-(3-dimethylaminopropyl) carbodiimide hydrochloride (EDC∙HCl) and N-hydroxysuccinimide (NHS) to yield mPEG–SS–NH_2_. ^1^H NMR and FTIR spectroscopy confirmed the successful synthesis of mPEG–SS–NH_2_.

The mPEG–SS–PZLL block copolymers were synthesized through ROP of ZLL NCA, initiated by mPEG–SS–NH_2_ in DMF. The successful synthesis of mPEG–SS–*b*–PZLL block copolymer was confirmed by ^1^H NMR, FTIR and SEC. The PDI values of the copolymers ranged between 1.09–1.14. Micelles were prepared by direct dissolution and dialysis methods. In the first, micelles were prepared by dissolving mPEG–SS–*b*–PZLL in PBS. It was found that the mPEG_113_–SS–*b*–PZLL_18_ had a spherical morphology with average radii around 130 nm and 181.0 ± 15.7 nm, as obtained by TEM and DLS measurements, respectively. Using the dialysis method, mPEG–SS–*b*–PZLL was dissolved in DMF, followed by addition of PBS. DMF was removed by dialysis against PBS. The hydrodynamic radius (Rh) was found to be 125.0 ± 5.9 nm and 103 nm by DLS and TEM measurements, respectively.

DOX-loaded mPEG–SS–*b*–PZLL micelles were prepared by a nanoprecipitation technique. The drug-loading abilities of the micelles were compared, and it was found that the higher DLC and DLE were associated with longer hydrophobic PZLL blocks. In the absence of GSH, less than 50 wt % loaded DOX was released from the micelles during the experiment (60 h). However, in the presence of GSH, DOX release rates accelerated, and over 60 h more than 90% of loaded DOX was released with 10.0 mM GSH, analogous to the intracellular reductive microenvironment. The rapid release of DOX from the micelles under reductive conditions could be attributed to the degradation of the micelles during cleavage of the disulfide bonds. The PZLL length was related to the drug release kinetics, and the DOX release rates from the micelles were on the order of mPEG_113_–SS–*b*–PZLL_18_ micelle (with 10.0 mM GSH) > mPEG_113_–SS–*b*–PZLL_35_ (with 10.0 mM GSH) > mPEG_113_–SS–*b*–PZLL_18_ micelle (without GSH) > mPEG_113_–SS–*b*–PZLL_35_ (without GSH). The decrease in the DOX release rate was a result of the increase in the DP of PZLL. Therefore, it was possible to modulate the DOX release rate from the micelles by adjusting the reductive environment and polypeptide content.

Li et al. [36] presented the synthesis of novel biocompatible and biodegradable shell-cross-linked micelles based on a lipoic acid (LA) decorated triblock copolymer resulting from the poly(ethylene glycol)–*b*–poly(*γ*-benzyl–l-glutamate)–*b*–poly(l-phenylalanine) (PEG–*b*–PBLG–*b*–PPhe) precursor. PEG–*b*–PBLG–b–PPhe triblock copolymers prepared by the one-pot, two-step sequential ROP of the monomers BLG–NCA and Phe–NCA initiated by *α*-methoxy-*ω*-amine-poly (ethylene glycol) (mPEG–NH_2_). PEG-*b*-PLG(EDA)–*b*–PPhe was synthesized through aminolysis with excess ethylenediamine (EDA). PEG–*b*–PLG(EDA-LA)–*b*–PPhe copolymers were obtained by amidation of PEG–*b*–PLG(EDA)–*b*–PPhe with lipoic acid in DMF in the presence of DCC and NHS. ^1^H NMR analysis was used to estimate the molecular characteristics of the terpolymers.

PEG–*b*–PGlu(EDA–LA)–*b*–PPhe self-assembled into micelles with the PEG as corona, PGlu(EDA–LA) in the middle shell and PPhe as the inner core. A 10% molar amount of DTT with respect to the lipoyl units was added at pH = 8.5, resulting in crosslinking after the reaction of lipoic acid with the small amount of DTT. Two series of micelles with encapsulated DOX were prepared, one with and one without the crosslinked middle shell. The DOX loading into the micelles included the slow addition of distilled water to a DMSO solution of PEG–*b*–PGlu(EDA–LA)–*b*–PPhe and DOX followed by dialysis against deionized water. Dynamic light scattering analysis was used to estimate the size of PEG–*b*–PGlu(EDA–LA)–*b*–PPhe micelles and the cross-linked micelles, i.e., 145.1 and 124.0 nm, respectively. Both types of micelle exhibited a spherical morphology as revealed by TEM analysis. In vitro drug release revealed that in 24h and under physiological conditions, approximately 36.4% and 41.4% DOX was released from DOX-loaded shell-cross-linked PEG–PGlu(EDA–LA)_10_–PPhe and PEG–PGlu(EDA–LA)_5_-PPhe micelles respectively, while in a reductive environment, DOX release was accelerated to 91.0% and 88.0%, respectively.

**Figure 11:**
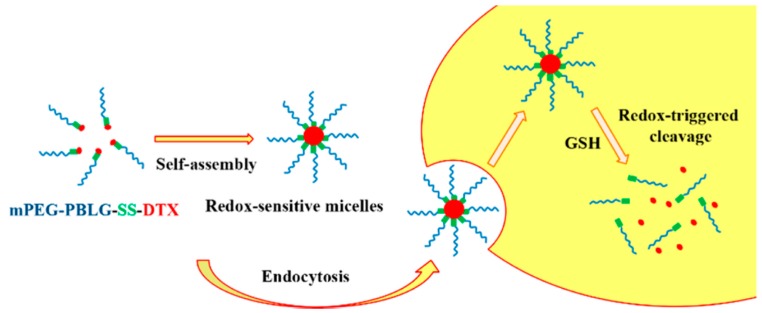
mPEG-PBLG–SS-DTXs Micelle Self-Assembly, Endocytosis by Tumor Cells, and Redox-Triggered Drug Release [37].

In 2016, Zhang et al. [37] reported the synthesis of a novel redox-sensitive drug delivery system based on poly(ethylene glycol)–*b*–poly(*γ*-benzyl l-glutamate)–SS–docetaxel (mPEG–*b*–PBLG–SS–DTX) conjugates. Copolymers of methoxy poly(ethylene glycol)–*b*–poly(*γ*-benzyl l-glutamate) (mPEG–PBLGs) with different molecular weights were synthesized via the ROP of BLG–NCA initiated by mPEG–NH_2_. In addition, 3,3′-thiodipropionic anhydride (DTDPA) was synthesized from the corresponding acid (DTDP) and reacted with the terminal amine group of mPEG–*b*–PBLG to result mPEG–*b*–PBLG–SS–COOH. The carboxyl group of mPEG–*b*–PBLG–SS-COOH was subsequently esterified with a hydroxyl group of DTX to afford the mPEG–*b*–PBLG-SS-DTX polymer with the conjugated drug. It was found that the copolymers self-assemble into nanosized micelles in aqueous environment via the dialysis method. The micelle sizes ranged between 101.3 and 148.9 nm, depending on the molecular weights and exhibited narrow size distribution, as obtained by TEM. The linkage containing a disulfide bond between the block polymer and DTX could be readily cleaved in the presence of reducing agent, such as DTT. Without DTT treatment, the release of DTX from the micelles was very slow, only 12.0% and 17.2% of DTX after 120 h. In contrast, the micelles showed very different release profiles in the presence of 50 mM DTT, where the release of DTX increased to approximately 40% after 120 h. These results suggest that the micelles as potential DDS, were stable in a nonreducing environment and could achieve rapid drug release upon cell internalization (Figure 11).

## 6. Photo-responsive Micelles

The synthesis of novel polypeptide micelles associated with indocyanine green (ICG) was reported by Wu et al. [38] in 2013 for both optical imaging and photothermal therapy of tumor. The authors took advantage of the exceptional photomechanical, photochemical and photobiological properties of ICG, and entrapped ICG in the core of a polymeric micelle. A triblock copolymer of poly(ethylene glycol)–*b*–poly(l-lysine)–*b*–poly(l-leucine) (PEG–*b*–PLL–*b*–PLLeu) was synthesized, and ICG was encapsulated through hydrophobic interactions in the hydrophobic core of PLLeu as well as through electrostatic interaction with the hydrophilic shell of PLL. The synthesis of the PEG–*b*–PLL–*b*–PLLeu micelle was performed via the ROP and sequential addition of the monomers methodology. PEG–NH_2_ was used as initiator and ZLL–NCA and LLeu–NCA as monomers for the block polymerization. The polymer self-assembled into micelles in aqueous media.

For the preparation of ICG-entrapped micelles, polymers and ICG were dissolved in DMSO, and the reaction mixture was dialyzed against the aqueous solution. DLS was used to estimate the dimensions, and TEM was employed to confirm the morphology of both ICG-free and ICG-loaded micelles. The ICG encapsulation efficiency (EE) was found to be 46.8% by fluorescence spectroscopy. It was shown that the micelle size was associated with the length of the PLL and PLLeu segments, and also that the cellular uptake efficiency could be controlled by altering these lengths. In this respect, in vitro and in vivo experiments revealed that PEG–*b*–PLL–*b*–PLLeu–ICG micelles had a high cellular uptake rate, special tumor targeting ability and prolonged circulation, rendering them ideal fluoroprobes for tumor diagnosis and imaging. In addition, PEG–*b*–PLL–*b*–PLLeu–ICG could be used as a photothermal agent for photothermal tumor therapies and cancer cell thermal ablation due to its good heating performance under NIR laser irradiation and successful in vitro photothermal cell ablation studies.

In 2016, Wu et al. [39] described the synthesis of plasmonic gold-embedded polypeptide micelles and their DOX-loaded counterparts under mild aqueous solution conditions. They synthesized a poly(cysteine)-*g*-poly(ethylene glycol) (PC–*g*–PEG) in three steps. The first step involved the ROP of the mixed monomers *S*-(*O*-nitrobenzyl)-l-cysteine *N*-carboxy anhydride (l-NBC–NCA) and D-NBC-NCA to form the poly(*S*-(*O*-nitrobenzyl)-d,l-cysteine) (PNBC). In the second step, the partial photocleavage reaction of PNBC solution took place after high-pressure mercury lamp irradiation at 365 nm, in order to cleave the NB groups. Dimethylphenylphosphine (DMPP) and poly(ethylene glycol) methyl ether acrylate (PEG acrylate) were then reacted with the thiol groups to form PNBC-*g*-PEG. The full photocleavage of NB groups was achieved with further UV irradiation to afford the final PC-*g*-PEG. ^1^H NMR and SEC analysis were used to calculate the number average molecular weight Mn and the rather high polydispersity value PDI (2.69) of the graft copolymers.

The procedure for the preparation of both gold-embedded micelles and their DOX-loaded counterparts requires two steps. In the first, the dialysis method was applied to form the uncrosslinked micelles and the DOX-loaded micelles of PC–*g*–PEG by a self-assembly process. Crosslinking was induced by the addition of a small amount of H_2_O_2_ in the solution. In the second step, the gold-embedded micelles resulted from the addition of HAuCl_4_ to the disulfide-bond-cross-linked micelle solution. Similarly, the DOX-loaded and gold-embedded micelles were formed by the addition of HAuCl_4_ to the DOX-loaded disulfide-bond-cross-linked micelle solution. The gold was reduced in situ and embedded into the interior core of the disulfide-bond-cross-linked polypeptide micelles through Au-S bonds. TEM and DLS analysis were used to determine the nearly spherical morphology of both micelles. The resulting gold-embedded polypeptide micelles revealed special chemical and photothermal properties and were biodegradable and biocompatible, with high photothermal conversion efficiency and good photostability detected from near infrared (NIR) analysis. In addition, in vitro and in vivo studies proved that the DOX-loaded and gold-embedded micelles presented a good synergistic chemo-photothermal effect, opening a new road in the evolution of anticancer treatment.

## 7. Multi-Responsive Micelles

In 2014, Guo et al. [40] reported the synthesis of a novel type of photosensitizer (PS)-loaded micelles. Cyanine dye was integrated in potential theranostic micelles for precise anatomical tumor localization via dual photoacoustic (PA)/near-infrared fluorescent (NIRF) imaging modalities, together with superior cancer therapy via sequential synergistic photothermal therapy (PTT)/photodynamic therapy (PDT). In order to produce this micellar system, authors synthesized the monomethoxy poly(ethylene glycol) and *n*-decylamine-grafted poly(l-aspartic acid) (mPEG–*b*–PAsp(DA)) copolymer. The synthetic procedure was performed by the ROP of BLA–NCA initiated by mPEG–NH_2_, followed by aminolysis of the *β*-benzyl groups by n-decylamine. The polymers were characterized by ^1^H NMR analysis which confirmed the degree of polymerization and the molecular characteristics of the copolymers. The photosensitizer clorin e6 (Ce 6) as well as the cyanine dye (Cypate) which exhibit enhanced photostability, cell internalization and tumor accumulation properties were employed and encapsulated into the micelles formed by the amphiphilic polymers.

The micelles containing Ce6 and cypate were formed by the nanoprecipitation methodology by dissolving the mPEG–*b*–PAsp(DA) with Ce6 and cypate in dimethyl sulfoxide (DMSO), followed by dialysis against de-ionized water. TEM was used to characterize the spherical morphology of the micelles, and DLS analysis was used to calculate the hydrodynamic diameter of the micelles at 63.6 nm. Due to the presence of PEG at the corona of the nanoparticles, enhanced stability in the blood compartment was expected. Due to the enhanced permeability and retention (EPR) effect as well as the large dimensions of the micelles, enhanced permeability and superior cancer cell internalization and accumulation properties were also observed. This type of micelle combined cancer diagnosis cancer along with its therapy. The dual photoacoustic/near-infrared fluorescent (NIRF) imaging modalities of the micelle revealed the high imaging contrast and spatial resolution of tumors, leading to definite anatomical localization of the tumor. At the same time, cancer treatment was achieved via sequential synergistic photothermal therapy/photodynamic therapy, as the micelles provoked selective photothermal damage on cancer cells that were accumulated.

The properties of star polymers, such as enhanced stability and high degree of drug encapsulation, prompted the synthesis by Wang et al. [41] of a novel core cross-linked dual responsive star polypeptide of the poly(l-glutamic acid)-*b*-poly(l-phenylalanine–*co*–l-cystine) [PLGA–*b*–P(l-Phe-*co*-l-CS)] type. The synthetic procedure involved the use of hexamethyldisilazane as the initiator for the ROP of the NCAs by the sequential addition methodology. First, the ROP of BLG-NCA was performed, followed by the copolymerization of Phe–NCA and Cys–NCA after completion of the polymerization of BLG-NCA. A star architecture was formed due to the crosslinking of Cys–NCA, a difunctional monomer, during its copolymerization with Phe–NCA. The deprotection of the benzyl group of the PBLG block was followed by treatment with a mixture of TFA and HBr 33% in acetic acid. After purification with dialysis and lyophilization, the blank micelles were formed by dissolving the polymers in DMSO followed by dialysis in water. The micelles were composed of PLGA as the outer corona and hydrophobic PLP/PLC as the inner core. The DOX-loaded micelles were formed by dissolving the polymer in water followed by addition of the solution of DOX in DMSO. Dialysis and freeze-drying followed for the removal of unentrapped DOX. For the preparation of the resveratrol (RES) -loaded micelles, the polymer and RES were dissolved in DMSO, and the solution was added to deionized water dropwise. After dialysis, filtration and lyophilizaton, RES-loaded micelles were obtained.

The size as well as the zeta potential of the blank micelles increased as pH was lowered. In the presence of 10 mM dithiothreitol (DTT), the micelle core was detached, resulting in an increase of their dimensions. SEM revealed that the micelles were spherical and that their size increased with drug loading. DLC and DLE values of DOX were higher than that of RES, due to the electrostatic attraction between the positively charged DOX and the carboxyl groups of PLG, compared to the aromatic stacking interaction of RES with poly(l-phenylalanine). The drug release curves revealed that the release of DOX was higher at acidic pH, while the release of RES was lower at the same conditions. Additionally, the release of RES was faster in the presence of 10 mM DTT.

Later, Gao et al. [42] reported the synthesis of a series of mixed shell micelles (MSMs) with approximately the same size, charge and core composition. The micelles were formed by the spontaneous self-assembly of various amphiphilic block copolymers. The polymers synthesized included poly(ethylene glycol)–*b*–poly(l-lysine) (PEG–*b*–PLL), PEG–*b*–(PLL–Tyr_1_), i.e., PEG–*b*–PLL with the terminal lysine capped with one tyrosine amino acid, poly(*N*-isopropylacrylamide)–*b*–poly(aspartic acid) (PNIPAM-*b*-PAsp), PAsp as well as PLL. The polymers resulted from the combination of ROP and ATRP. The PEG–*b*–PLL copolymer was synthesized by the ROP of ZLL–NCA of the amine end-functionalized PEG, followed by deprotection. PEG–*b*–(PLL–Tyr_1_) was synthesized by reaction of the terminal amine groups with tyrosine. PNIPAM-NH_2_ was synthesized by the ATRP of *N*-isopropylacrylamide with *t*-Boc-aminoethyl 2-bromoisobutyrate as the initiator, followed by deprotection of the Boc groups. PNIPAM–*b*–PAsp was synthesized with PNIPAM–NH_2_ as the macroinitiator of the ROP of *β*-benzyl l-aspartate-*N*-carboxyanhydride, followed by deprotection. The homopolypeptides were synthesized using *n*-butylamine as the initiator for the ROP of the corresponding *α*-amino acid NCAs, followed by deprotection. ^1^H NMR measurements confirmed the successful syntheses, and the molecular weights were measured by SEC.

The mixed micelles were formed by combining PEG_113_–*b*–PLys_40_ and PNIPAM_80_–*b*–PAsp_43_ in four different weight ratios of the PEG / PNIPAM segments (*W*_PEG_/*W*_PNIPAM_) equal to 10/0, 7/3, 5/5, and 3/7, and were labeled MSMs-0, MSMs-30, MSMs-50, and MSMs-70. The N/C (amine/carboxylate) ratio was kept at 1 for each solution by adding homopolymer PAsp_42_ or PLL_18_ in order to obtain a slightly negative charge reducing the nonspecific uptake by liver and spleen due to the electrostatic repulsion between negatively charged micelles and cellular surface.

For the synthesis of MSMs-0, only block copolymer PEG–*b*–PLys and homopolymer PAsp were employed. The crosslinking between the –NH_2_ of PLL and the –COOH of PAsp occurred in the presence of EDC·HCl. Micelles were characterized DLS and TEM. The MSMs possessed similar uniformity and nanosize of approximately 100 nm. MSM stability was estimated by DLS in 10 mM PBS buffer in a physiological concentration of bovine serum albumin (BSA). Over 48 h, the MSM size distribution remained at approximately 100 nm, and after incubation at 37 °C no apparent size variation was observed for MSMs-0, MSMs-30 or MSMs-50, indicating that these MSMs remain stable under these conditions. However, size variation was observed for MSMs-70 in the presence of BSA at 48 h. The intensity of the MSMs-70 around 100 nm decreased, and the size distribution broadened at 48 h. The slight aggregation of MSMs-70 might be attributed to the large hydrophobic area exposed on the micelle that interacted hydrophobically with the BSA. Not only long blood circulation of the nanocarriers was achieved, but their deposition in liver and spleen also decreased.

In addition to the entrapment of anticancer drugs within nanoparticles for the selective targeting and subsequent killing of cancer cells, micelles containing photosensitizing molecules which allow for photodynamic therapy or diagnosis of cancer cells have also been presented. Liu et al. [43] reported the synthesis of the pH-responsive copolymer poly[oligo(ethylene glycol) methacrylate]–*b*–poly(diisopropylethylamine l-aspartamide) (POEGMA–*b*–PAsp(DIPEA)), for the encapsulation of the photosesnsitizing molecule 3,7-dibromo-2,8-di(4-methoxyphenyl)-11-trifluoromethyl-dithieno[2,3-b]-[3,2-g]-5,5-difluoro-5-bora-3a,4a-diaza-s-indacene (BODIPY–Br_2_).

The synthetic approach of the polymer involved the synthesis of each block separately, followed by the coupling with click chemistry. Propargylamine was used for the ROP of BLA–NCA to afford an *α*-end functionalized PBLA featuring a triple bond. The POEGMA block that was end-functionalized with an azide group was synthesized using a RAFT agent as initiator featuring an azide group for the polymerization of oligo(ethylene glycol) methacrylate. The two blocks were then linked by click chemistry. Finally, diisopropylethylamine (DIPEA) was conjugated to PAsp through an amide bond formation, to afford POEGMA–*b*–PAsp(DIPEA).

This polymer formed micelles with POEGMA forming the hydrophilic shell and PAsp(DIPEA) in the hydrophobic core for the loading of the anticancer drug DOX or the photosensitizer BODIPY-Br_2_. The photosensitizer has superior properties as imaging agent, e.g., high photo-stability, fluorescence quantum yield and extinction coefficient compared to other dyes, and therefore has been used in numerous applications such as imaging and photodynamic therapy. The micelles with entrapped DOX or BODIPY-Br_2_ were fabricated through the nanoprecipitation methodology, i.e., the polymer and DOX or BODIPY-Br_2_ were dissolved in DMF, triethylamine was added and deionized water was dropped into the solution, which was dialyzed against ultrapure water. The photosensitizer loading content was 2.02% while the DLC was 1.04%, though the efficiency was 13.2% and 11.4%, respectively. The drug release at pH 7.4 was slow, at a total value of 27%, while at pH 5.5 was faster and sustaining for 96 h, with 60% total drug release. At pH 5.5, BODIPY-Br_2_ formed precipitates. DLS measurements revealed that the micelles loaded with the photosensitizer are slightly smaller at pH = 7.4 than the blank micelles, verifying the existence of hydrophobic and electrostatic interactions between polymer and photosensitizer. At pH 5.5, the micelles became swollen as determined by DLS and AFM.

Yang et al. [44] designed redox and pH dual-sensitive biodegradable micelles based on disulfide-linked methoxy poly(ethylene glycol)–*b*–poly[2-(dibutylamino)ethylamine-l-glutamate] (mPEG–SS–PNLG) copolymer as a smart carrier for intracellular DOX release. They initially synthesized the macroinitiator methoxy-poly(ethylene glycol)-cystamine (mPEG–SS–NH_2_). mPEG–OH, pyridine and 4-nitrophenyl chloroformate were then employed for the activation of mPEG, followed by esterification with cystamine∙2HCl and triethylamine in order to obtain the final ester. Subsequently, mPEG–SS–NH_2_ was used as the macroinitiator for the ROP of BLG–NCA to afford the diblock copolymer mPEG–SS–PBLG. Partial aminolysis of the benzyl protective groups was achieved by reaction with 2-(dibutylamino)ethylamine (10× mol ratio to the benzyl groups of mPEG–SS–PBLG) and 2-hydroxypyridine (5× mol ratio to the benzyl groups of mPEG–SS–PBLG) to give the mPEG–SS–PNLG terpolymer, containing partially grafted 2-(dibutylamino)ethylamine moieties, carboxyl groups of the mPEG–SS–PBLG precursor through debenzylation reaction. It was found that the substitution of benzyl groups can be controlled by varying the reaction time or the feed molar ratio of 2-(dibutylamino)ethylamine to benzyl groups. The solubility of mPEG–SS–PNLG copolymers was also related to the percentage of benzyl groups converted. Addition of PBS and adjustment of pH at 7.4 with 0.1 M NaOH was used for the micelle production. The micelles were loaded with DOX, after dissolving the polymer in THF followed by dialysis. Titration experiments revealed that the pKa values of mPEG–SS–PNLG copolymers increased as the conversion of benzyl groups became higher due to hydrophobicity of benzyl groups rather than that of the 2-(dibutylamino)ethyl groups. Lowering the pH resulted in an increase in the size of the nanostructures because the micelles swelled due to the protonation of tertiary amine groups rendering the micelle core less hydrophobic. The disulfide bonds in the mPEG–SS–PNLG micelles were disrupted as 10 mM DTT at pH 7.4 was used, as confirmed by DLS since the size of the aggregates increased. Drug release experiments suggested that lower pH values and larger GSH concentrations led to an increase of the DOX release rate. In vivo and in vitro experiments showed that the empty polymeric micelles are non-toxic to HepG2 cells and the DOX-loaded mPEG–SS–PNLG micelles possess efficient apoptosis.

In 2017, Gao et al. [45] reported the synthesis of reduction-thermo-sensitive, sillica-cross-linked polypeptide hybrid micelles (CCMs). Trimethoxysilane-terminated and disulfide-bond-centered polyglutamate homopolymers with terminal trimethoxysilane groups were synthesized. The synthetic approach first involved the utilization of cystamine as the difunctional initiator for the ROP of BLG, followed by reaction of the terminal amine group with the epoxy group of *γ*-(2,3-epoxypropoxy) propytrimethoxysilane. The micelles were prepared through the sol-gel cross-linking reaction of the trimethoxysilane in alkaline aqueous solution to afford the CCMs, followed by dialysis for purification. The same methodology was used to load DOX into the micelles. DLS and TEM employed for the examination of the characteristics of the micelles revealed that CCMs have smaller size and flatter morphology in comparison with the non-crosslinked micelles. It was determined that the sol-gel cross-linking mainly occurred within the inner core of micelles and not in inter-micelles. DTT was used for reduction of the non-crosslinked micelles, while the size of CCMs remained the same over 48 h in the presence of DTT, due to the Si–O–Si covalent bonds. Both non-cross-linked micelles and the CCMs show a reversible thermo-sensitivity in aqueous solution, as presented by the results of heating-cooling cycles.

DOX-loaded CCMs showed a much lower fluorescence intensity in contrast to free DOX at same concentration, proving that DOX was encapsulated in the hydrophobic region of the micelles. Both DOX-loaded non-cross-linked micelles and CCMs adopted spherical morphology. The DOX-loaded micelles are larger in size compared to the blank micelles, further verifying DOX encapsulation into the micelles. In vitro drug release experiments were conducted in PBS at pH 7.4 and 37 °C with and without addition of 10 mM DTT. The DOX-loaded CCMs showed reduction-triggered drug release properties in comparison to non-cross-linked micelles and could be quickly internalized by HeLa cell.

## 8. Polypeptide-Based Micelles Bearing a Surface Ligand

An optimized, pH-sensitive mixed-micelle system conjugated with folic acid was prepared in order to challenge multidrug resistance (MDR) [46]. The polymers that were synthesized were of the poly(l-histidine-*co*-l-phenylalanine)–*b*–poly(ethylene glycol) (PEG) type blended with poly(l-lactic acid)–*b*–PEG–folate (PLLA–*b*–PEG–folate). The mixtures formed pH-sensitive micelles due to the presence of poly(l-histidine), and the pH value that the micelles were disrupted was controlled by the copolymer composition and weight ratio between the two copolymers. Micelles formed by the mixtures were fine tuned to be disrupted at early endosomal pH. The preparation of the copolypeptides involved the random ring opening copolymerization of the benzyl protected l-histidine (Bz–His–NCA) and l-phenylalanine NCA (Phe–NCA), initiated by isopropylamine at various feeding ratios. The coupling reaction of the resulting PBHP with PEG was carried out with monocarboxylated PEG, activated with NHS and DCC. Finally, the benzyl protective groups of poly(l-histidine) were removed from the imidazole ring with anhydrous liquid ammonia and finely cut metallic sodium. The molecular weight of the copolymers was estimated by ^1^H NMR. The PLLA–*b*–PEG diblock copolymer was synthesized by ROP of l-lactide initiated by hydroxy group of PEG monoacid in the presence of stannous octoate as a catalyst at 60 °C. PLLA-*b*-PEG–folate was prepared by conjugation of folate–NH_2_ with activated PLLA–*b*–PEG–COOH.

The micelles formed were composed of a hydrophobic core containing histidine/phenylalanine (16 mol %) copolypeptide (80 wt %) and poly(l-lactic acid) (20 wt %). DOX was entrapped within the micellar core by the nanoprecipitation methodology. The optimum polymer composition was obtained by optimization of the micelle properties in the presence of ≈ 20 wt % DOX. In order to evaluate the pH sensitivity of the micelles, critical micelle concentrations (CMC), micelle size, and transmittance of micelle solutions were measured as a function of pH. It was found that the size of the micelles with DOX increased at pH values below ~6.5, and the release curves indicate increasing drug release at lower pH (5.5) rather than at physiological pH (7.4).

The pH-dependent cell viability was tested against ovarian carcinoma cell lines (A2780 wild-type and DOX-resistant counterpart, A2780/DOX) that overexpress the folate receptor on the cell surface in order to compare the effect of each formulation on the cells. Free DOX and DOX encapsulated in pH-insensitive PLLA–*b*–PEG micelles with folate (DOX/PHIM-f) or without folate (DOX/PHIM) were employed as controls. It was found that mixed micelles without folate conjugation showed a higher toxicity at pH 6 and 5.5. In order to avoid the overlapping effects of Lysotacker red with DOX fluorescence, the green-fluorescence dye *N*-(5-dimethylaminoapthalene-1-sulfonyl)-1,2,-dihexadecanoyl-Sn-glycero-3-phosphoethanolamine (DHPE) was used as a marker for the intracellular distribution as well as to demonstrate that the drug loaded on the pH-sensitive mixed micelle escapes from the endosomal compartment. DHPE fluorescence does not significantly overlap with endolysosomal compartments and is observable in the nucleus. Upon encapsulation of DOX, the cytofluorometry obtained from MDR cells indicated that the pH-sensitive mixed micelle with folate was associated with a 3–4 fold higher DOX intensity than that of pH-insensitive micelles with folate.

**Figure 12:**
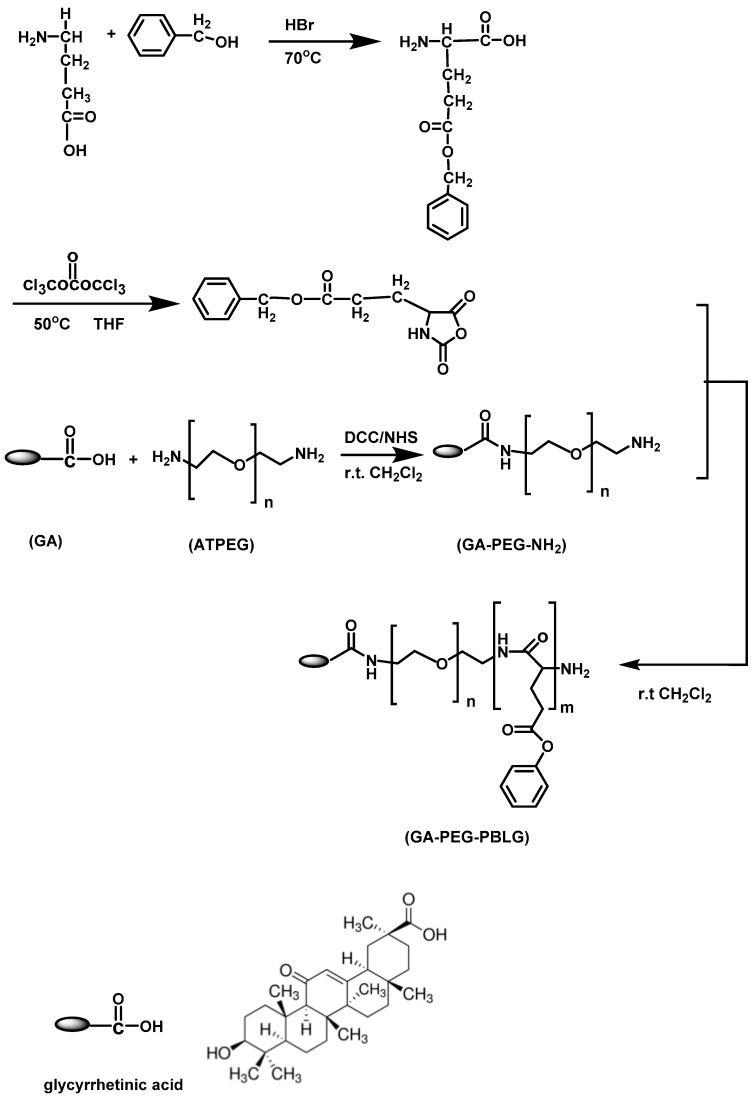
The reactions used for the synthesis of GA–PEG–PBLG block copolymers.

In another work, Huang et al. [47] reported the synthesis of glycyrrhetinic acid-modified poly(ethylene glycol)-*b*-poly(*γ*-benzyl-l-glutamate) micelles for liver targeting therapy. A two-step procedure was used for the synthesis of the copolymers. Initially, *α*-glycyrrhetinic acid, *ω*-amine end-functionalized poly(ethylene glycol)-(GA–PEG–NH_2_) was synthesized and utilized as a macroinitiator of the ROP of BLG–NCA. The macroinitiator was synthesized by reacting glycyrrhetinic acid with poly(ethylene glycol)-bis-amine in the presence of DCC as well as *N*-hydroxysuccinimide. A thin ninhydrin assay was employed to confirm the absence of unreacted GA–PEG–NH_2_. PEG-*b*-PBLG block copolymers without glycyrrhetinic acid were also synthesized by the ROP of BLG–NCA using mPEG–NH_2_ as a macro-initiator (Figure 12). FT-IR and ^1^H NMR were used to confirm the successful synthesis. The polymers formed micelles in aqueous solutions, and their average size and size distribution were monitored by DLS measurement. It was found that the micelle size increased with decreasing PEG length. The preparation of DOX-loaded micelles by the nanoprecipitation method followed. Block co-polymer and DOX–HCl were dissolved in DMF in the presence of TEA and were then stirred at room temperature until all components were dissolved. To form drug-loaded micelles, a dialysis bag containing drug solution was suspended in acetate buffer, and the system was dialyzed against deionized water. The morphology of the resulting micelles was observed by TEM. DLS was employed to determine the hydrodynamic diameter (Dh) and PDI of the micelles.

It was found that DOX/GA–PEG–*b*–PBLG micelles had a regular spherical shape. The diameters of GA–PEG-*b*-PBLG, DOX/GA–PEG–*b*–PBLG, PEG–*b*–PBLG and DOX/PEG–*b*–PBLG were calculated at 181.8, 214.2, 175.4 and 206.6 nm, respectively. The DOX release profiles from both GA–PEG–*b*–PBLG and PEG-*b*-PBLG micelles were similar at the same pH, indicating that the introduction of GA did not change the DOX release. At pH 7.4 after an initial drug burst of about 20 wt % in one day, a plateau was observed for the release of DOX in the following 6 days with a cumulative release of 26 wt %. At pH 5.8, the release rate was rapid and more than 45 wt % of DOX was released on day 1, followed by a constant slow release which approached 60 wt % over the next 6 days. The pH-dependent release behavior could result from the protonation of the DOX amino group under acidic conditions, resulting in the diminished hydrophobic interaction between DOX and the PBLG segment. Based on these observations, only a small amount of DOX is expected to be released from the micelles in the systemic circulation (pH 7.4), reducing the side-effects and heart cytotoxicity caused by DOX leakage. However, a more rapid release should occur once the micelles reach the tumor site, due to the lower pH environment (pH 5.8). The cellular uptake investigation revealed that the GA introduction to the micelles could significantly increase their affinity for human hepatic carcinoma 7703 cells, since a 3.7-fold higher endocytosis compared to that of unmodified micelles was observed.

Later, the same group [48] used the same polymeric nanoparticles to perform similar studies as liver targeted drug carriers and found similar results.

Nanoparticles bearing a special functional group at the end of the macromolecule can overcome MDR. Li et al. [49] synthesized novel amphiphilic copolymers composed of heparin as the main chain grafted with PBLA and conjugated with folate or grafted with folate functionalized poly(ethylene glycol). For the synthesis of the PBLA the BLA–NCA was first synthesized. The PBLA was synthesized by the ROP of BLA–NCA using butylamine as initiator. FA–NH_2_ was synthesized by reacting folic acid with ethylenediamine in the presence of DCC and NHS, while folate end-functionalized PEG was synthesized by reacting folic acid with PEG-bis amine, in the presence of DCC and NHS. Finally, heparin was grafted to PBLA using the coupling agent EDC or, in the case of folate conjugated amphiphilic copolymers, FA–PEG was added first in the PBLA solution followed by the addition of the heparin solution with EDC. All structures were characterized by ^1^HNMR and UV and the nanoparticles were analyzed by dynamic light scattering. The CMC of the heparin-based copolymers was determined by fluorescence spectrometry and the morphology of the nanoparticles was observed by field emission scanning electron microscope (SEM).

The hyperbranched polyester polymer Boltorn H40 bearing 64 hydroxyl terminal groups per molecule was used as a core material for the synthesis of a hyperbranched amphiphilic polymer bearing multiple R-PEG-*b*-PLGA(DOX) chains [50]. The moiety R is either cyclo(Arg-Gly-Asp-D-Phe-Cys) peptide or macrocyclic chelators (1,4,7-triazacyclononane-*N,N’*,*N*’’-triacetic acid) [NOTA] in the outer periphery, while DOX is chemically linked at the carboxyl groups of the PLGA through acid labile hydrazine bonds. The multiarmed hyperbranched material forms unimolecular micelles in aqueous solutions used for cancer-targeted drug delivery and non-invasive positron emission tomography (PET) imaging in tumor-bearing mice.

The synthetic approach involved the synthesis of PBLG-*b*-PEG–Mal and PBLG–*b*–PEG–OCH_3_ block copolymers by ROP of BLG–NCA, using Mal–PEG–NH_2_ or OCH_3_–PEGNH_2_ as macroinitiators, respectively. Mal, a maleimido group, is used for the end-functionalization of the final polymer with two different surface markers to target cancer cells. The hydroxyl groups of H40 were then transformed to carboxyl groups by reaction with succinic anhydride, followed by reaction of these groups with the amine of PBLG–*b*–PEG–Mal or PBLG-*b*-PEG–OCH_3_ to afford the hyperbranched amphiphilic H40-PBLG–*b*–PEG–OCH_3_/Mal copolymer. The Mal terminal groups present in PEG–Mal were used for cRGD and NOTA conjugation, and the -OCH_3_ groups present in PEG–OCH_3_ were used to help control the molar ratio of maleimide at the micelle surface, which subsequently controls the density of cRGD and NOTA.

In a following two-step reaction, DOX was conjugated to the H40-PBLG*-b-*PEG–OCH_3_/Mal polymer. The H40–PBLG–*b–*PEG–OCH_3_/Mal benzyl groups were substituted with hydrazide groups to give H40*–*P(LG*–*hydrazide)-*b*-PEG-OCH_3_/Mal by an ester-amide exchange aminolysis (EAE) reaction. An excess amount of DOX was subsequently conjugated to the H40*–*P(LG*–*hydrazide)*-b-*PEG*–*OCH_3_/Mal hydrazide group with a pH-sensitive hydrazine linkage. Finally, the cRGD peptide for integrin avb3-targeting and NOTA for 64Cu chelation were conjugated onto the surface of the H40*–*P(LG*–*Hyd*–*DOX)*–b–*PEG–OCH_3_/Mal copolymer through reaction between the Mal groups of H40*–*P(LG*–*Hyd*–*DOX)*–b–*PEG–OCH_3_/Mal with the thiol groups of cRGD and/or thiolated NOTA (NOTA*–*SH) to afford the final product H40*–*P(LGHyd-DOX)*–b–*PEG–OCH_3_/cRGD/NOTA.

**Figure 13:**
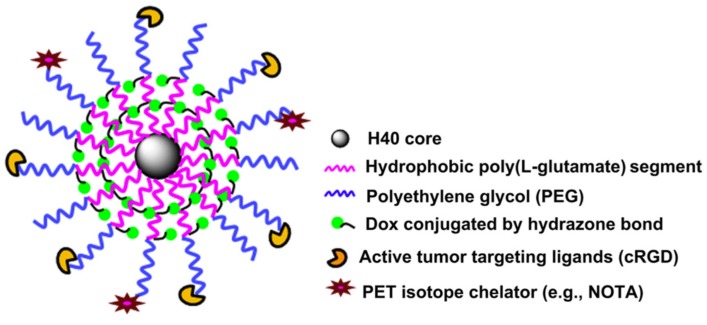
H40-DOX-cRGD star-shaped nanocarriers for tumor-targeted drug delivery and PET imaging [50].

The hyperbranched amphiphilic block copolymer H40*–*P(LG*–*Hyd*–*DOX)*–b–*PEG–OCH_3_/cRGD/NOTA forms a stable unimolecular micelle in an aqueous solution due to the large number of amphiphilic arms (w25) with appropriate hydrophilic to hydrophobic ratios and to its globular architecture (Figure 13). The hydrodynamic diameter of the H40*–*DOXcRGD micelles ranged from 44 to 91 nm, with an average diameter of 65 nm as measured by DLS. TEM images showed that the unimolecular micelles possessed a spherical shape with diameters ranging from 22 to 31 nm. In order to investigate the pH-sensitivity of H40*–*DOX*–*cRGD, in vitro drug release studies were carried out under simulated physiological and cellular conditions at pH 5.3, 6.6, and 7.4 at 37 °C. The pH of the medium strongly influenced the DOX release rate from H40*–*DOX*–*cRGD. Over the pH range of 5.3–7.4, the release rate increased consistently. At pH 7.4, the amount of DOX released after 45 h was 12.1%, including an initial burst release of about 4.7%. However, after 45 h and at pH values of 5.3 and 6.6, the amount of DOX released was approximately 92.7% and 85.6%, respectively, verifying that DOX release from H40*–*DOX*–*cRGD in an acidic environment was controlled by the acid-cleavable hydrazone linkage.

It has been proven that polymers with galactosyl group are attractive for targeted intracellular antitumor drug delivery to hepatoma cells through conjugation with asialoglycoprotein receptor (ASGP-R). Using this function, Ding et al. [51] synthesized poly(OEGylated l-glutamate)-*b*-poly(l-glutamic acid) (PMLG*-b-*PLGA) (OEG, i.e., oligo(ethylene glycol)) and poly(galactosylated l-glutamate)-*b*-poly(l-glutamic acid) (PGLG*-b-*PLGA) in a four-step procedure. Initially, PPLG was synthesized via ROP of PLG–NCA utilizing *n*-hexylamine as the initiator. Then, PPLG was used as the macroinitiator for the ROP of BLG–NCA resulting in the copolymer PPLG*–b–*PBLG. Following deprotection of the benzyl group with dichloroacetic acid and HBr/acetic acid, PPLG*–b–*PLGA resulted, and reaction with 1-azido-2-(2-(2-methoxyethoxy)ethoxy)ethane (MEO_3_-N_3_) or 2′-azidoethyl-*O*-*β*-d-galactopyranoside (galactose-N_3_) in the presence of CuSO_4_/NaAsc gave PMLG-*b*-PLGA or PGLG*-b-*PLGA, respectively.

DOX-loaded PMLG*–b–*PLGA and PGLG*–b–*PLGA micelles were prepared by a dialysis technique by mixing PMLG*–b–*PLGA or PGLG*–b–*PLGA and DOX·HCl in PBS at pH 7.4. PBS was removed by dialysis against deionized water. To determine drug loading content and drug loading efficiency, the product was dissolved in *N*,*N*-dimethylformamide (DMF) and analyzed with fluorescence. The hepatoma-targeted nanomedicine was prepared by cooperative self-assembly of galactopeptide and DOX induced by two-stage physical interactions. In the cooperative self-assembly strategy, the carboxyl- side groups were utilized to encapsulate DOX through electrostatic interaction and/or hydrogen bond formation with its amine group. The amphiphile then self-assembled into micellar nanoarchitecture induced by the hydrophobic (e.g., *π–π*) interaction between the DOX moieties. Both PMLG_7_*–b–*PLGA_22_ and PGLG_7_*–b–*PLGA_22_ showed a spherical morphology with the respective average radii of approximately 30 and 50 nm, respectively.

The Rh value of the loaded nanoparticle was also affected by the copolypeptide composition. Longer PLGA blocks resulted in lower Rh values, because of the increased hydrophobic interactions between the DOX-conjugated PLGA chains. The DOX release rate increased as the pH decreased from 7.4 to 5.3. This pH-dependent behavior is probably the result of a decrease in the PLGA*–*DOX interaction upon instantaneous protonation of the carboxyl groups and DOX in the core at low pH. In addition, the copolypeptide composition could be correlated to the drug release kinetics, and the release rate was as follows: PGLG_24_*–b–*PLGA_9_ > PMLG_24_*-b-*PLGA_9_ ≫ PGLG_7_*-b-*PLGA_22_ > PMLG_7_*–b–*PLGA_22_ ≫ PGLG_10_*–b–*PLGA_48_ > PMLG_10_*–b–*PLGA_48_ at all the pH values. In addition, the slightly faster DOX release from galactosylated nanomedicine compared with that of OEGylated nanomedicine could be due to the stronger affinity between galactosyl group and water.

In a more recent work, Qian et al. [52] reported the synthesis of a folate-decorated 3-arm star-block terpolymer of the [poly(*γ*-hydrazide–l-glutamic acid)–*b*–(poly(*N,N*-dimethylaminopropyl methacrylamide)-*g*-poly(ethylene glycol/folic acid))]_3_ ([PGAH–*b*–(PDMAPMA-*g*-FA/PEG)]_3_) type as a novel nanocarrier for the targeted co-delivery of DOX and Bcl-2 siRNA to breast tumor tissue. FA/PEG is PEG end-functionalized by folic acid. The terpolymer was prepared with a combination of different reactions such as ROP, RAFT, PEGylation and hydrazinolysis.

Initially, three-arm star-shaped polypeptide (PBLG)_3_ was synthesized through the ROP of BLG-NCA initiated by the trifunctional initiator tri(2-aminoethyl) amine (TAEA). The primary amino end groups of (PBLG)_3_ were then reacted with the carboxyl groups of S-1-dodecyl-S’-(*α,α’*-dimethyl-*α*’’-acetic acid) trithiocarbonate (DDACT) via carbodiimide reaction using N,N’-dicyclohexyl carbodiimide (DCC) as a coupling agent to form (PBLG)_3_ macro-RAFT agent. The synthesis of the three-arm star-shaped diblock polymer (PBLG–*b*–PDMAPMA)_3_ occurred by RAFT copolymerization of *N*,*N*-dimethylaminopropyl methacrylamide (DMAPMA) and N-methylol acrylamide (NMAm) initiated by 2,2’-azo-bis(isobutyronitrile) (AIBN). For the conjunction of folic acid (FA) onto one end of the PEG chain, bis-isocyanate-terminated PEG (ONC–PEG–CNO) was first prepared with the reaction of bis(*β*-isocyanatoethyl) disulfide (BIED) with HO–PEG–OH. The OCN–PEG–NCO reacted with FA to yield FA–PEG–NCO. (PBLG–*b*–(PDMAPMA-*g*-FA/mPEG))_3_ was formed via carbamation reaction between the pendant hydroxyl groups of PDMAPMA blocks in (PBLG–*b*–PDMAPMA)_3_ and the isocyanate end groups of FA-PEG-NCO and mPEG-NCO. The final terpolymer (PGAH–*b*–(PDMAPMA-*g*-PEG))_3_ was prepared through a hydrazinolysis reaction, when the pendant benzyl protected groups of PBLG blocks were converted into hydrazide groups.

DOX was conjugated with PGAH*–b–*(PDMAPMA-*g*-PEG)_3_ terpolymer via an acid-labile hydrazone linkage, and siRNA was attached through electrostatic interaction with the dimethylamino groups, to afford “two-in-one” nanomicelleplexes in aqueous solutions with a spherical shape and a size of 101.3 nm. As DLS and TEM analysis showed. The formation of this type of micelles included PGAH as the core, PDMAPMA as the inner shell and PEG as the stealth outer corona. FA was used as the targeting moiety on nanomicelles because of its high affinity to folate receptors which are overexpressed in many tumors. The DOX release rate was studied, and DOX was found to be released faster at acid pH 5.0 than at normal pH 7.4, indicating that this terpolymer has promising properties as a new drug delivery system for cancer treatment.

In 2014 Shao et al. [53] reported the development of a smart therapeutic nanodevice with cooperative dual characteristics of high tumor-targeting ability and selective control of drug deposition in tumor cells. This nanodevice was prepared with a crosslinker containing a disulfide linkage to form an inner cellular microenvironment-responsive “–SS–” barrier. The polypeptide-containing terpolymers CH_3_O*–*PEG*–b–*PLL*–b–*PPhe or N_3_*–*PEG–*b*–PLL–*b*–PPhe, were synthesized via a two-step ROP in the presence of CH_3_O–PEG–NH_2_ or N_3_–PEG–NH_2_ as the initiator and sequential addition of the monomers, ZLL–NCA first and Phe–NCA after completion of polymerization.

The synthesis of the macroinitiator featuring one azide and one primary amine groups at the two ends of the chain was achieved by the conversion of hydroxy of HO–PEG–NH_2_ to an azide to give N_3_–PEG–NH_2_. This transformation was performed by reaction of an *α,ω*-dihydroxy end-functionalized PEG with 2.2 equiv of methane sulfonyl chloride in the presence of triethylamine as base, followed by reaction with 2.5 equivalents of sodium azide. The crucial monoreduction step was achieved by reaction of the bisazide with 1.05 equivalents of triphenylphosphine. After deprotection of PZLL blocks, the polymers that contained a terminal azide group (N_3_–PEG–*b*–PLL–*b*–PPhe) was conjugated with dehydroascorbic acid (DHA) featuring a propargylic group via click reaction. In this work, four kinds of polymeric micelles were prepared: DHA-modified cross-linked or non-cross-linked micelles loaded with PTX (DHA–PEG–*b*–PLL(SS)-*b*-Phe/PTX or PEG–*b*–PLL–*b*–Phe/PTX) and unmodified cross-linked or non-crosslinked micelles loaded with PTX (PEG–*b*–PLL(SS)–*b*–Phe or PEG–*b*–PLL–*b*–Phe/PTX). To build the disulfide linkage, the amine groups of PLL were reacted with the difunctional reagent 3,3’-dithiobis(sulfosuccinimidylpropionate) (DTSSP), featuring NHS groups. The encapsulation of PTX was performed by the nanoprecipitation methodology, resulting in the formation of core-shell micelles. The crosslinking of the micelles was performed after drug encapsulation.

The drug-loaded nanodevice should remain intact in the bloodstream. Once in the tumor cells, the high level of intracellular glutathione triggered drug release. The combination of these two features were combined, the smart nanodevice significantly improved the tumor-targeting and drug-delivery efficiency. The synthesized micelles displayed better deep penetrating ability and accumulation in tumors due to their small particle size. This is an important advantage to treat glioma, where the pore size of fenestrated capillary walls is smaller than in other tumors and restricts larger particle leakiness. DHA actively directed micelle accumulation in the tumor site via the overexpressed GLUT1 binding. Additionally, drug delivery was controlled through the disulfide bond cross-linked barrier, which could protect drug leakage against blood dilution. The micelles featuring the crosslinks became much more condensed after cross-linking. When the micelles are inserted in the medium of GSH-free or low GSH concentration, crosslinked micelles remained stable with only 20% of PTX release within 48 h, while as the GSH concentration increased up to the cytoplasm level, the release markedly increased.

To evaluate the glioma-targeting effect, the micelles were i.v. injected into glioma-bearing mice. The micelles featuring DHA groups exhibited much higher targeting efficiency in glioma sites than those without this group, and the crosslinked micelles had a slower release profile as compared to those without crosslinks.

**Figure 14:**
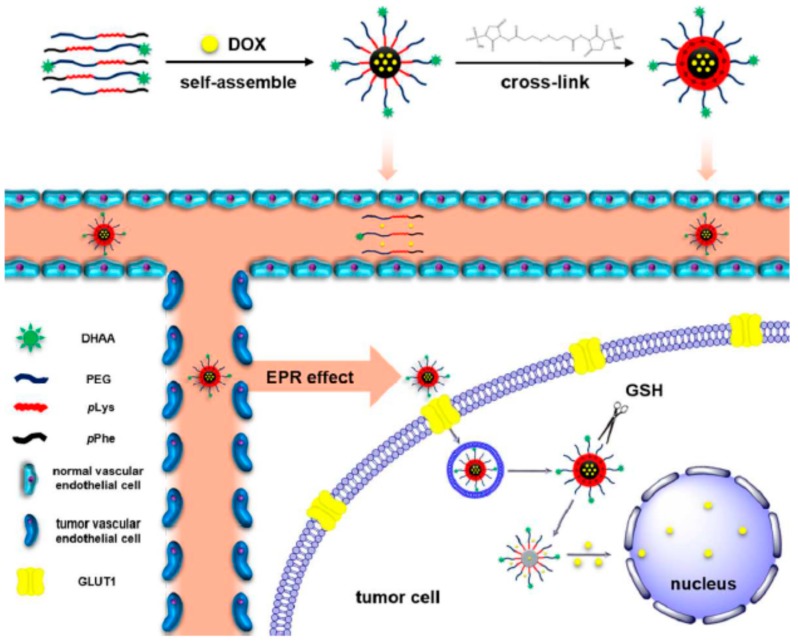
Stepwise synthesis, GLUT1-mediated endocytosis and GSH-triggered intracellular drug release of DPL(SS)P/DOX micelles [54].

One year later, the same group [54] reported the synthesis of similar polymers featuring the dehydroascorbic acid (DHA) that formed micelles modified with disulfide cross-links for the hepatocellular carcinoma (HCC) treatment. These micelles were consisted of DHA–PEG–*b*–PLL(SS)–*b*–Phe polymers, in which the amino groups of PLL were crosslinked. In this work, DOX was encapsulated. The synthesis of the polymers was similar to that published by Shao et al. [50]. The final polymers were characterized by nuclear magnetic resonance ^1^H NMR analysis. DHAA which was attached in the surface of micelles enhanced the accumulation in target cells due to its exceptional properties, as a substrate of GLUT1, a member of glucose transporter family overexpressed on hepatocarcinoma cells. The encapsulation of DOX was performed by the nanoprecipitation method, by dissolving the polymers and the drug in DMF followed by dialysis against deionized water. Crosslinked polymers/DOX micelles were obtained by the addition DTSSP, DLS analysis showed that the average hydrodynamic diameter of uncrosslinked polymer/DOX micelles exhibited 60.3 nm diameter, while the crosslinked polymer/DOX micelles were 52 nm. The spherical morphology of both types of micelles was confirmed using AFM. In vitro DOX release occurred via dialysis method, where it was shown that for the crosslinked polymers 20% of DOX was released in 48 h, while the uncrosslinked polymers presented 30% at the same time. DOX release was also investigated in the presence of a high intracellular GSH concentration. The studies showed that DOX release from the crosslinked polymer/DOX micelles was accelerated, as 40% of DOX was released within 2 h and 70% after 48 h, due to the cleavage of disulfide linkages when exposed to cytoplasm level of GSH (Figure 14).

In a recent work Miura et al. reported the synthesis of a novel block copolymer poly(ethylene glycol)-*b*-poly(*γ*-benzyl–l-glutamate), where PEG is end-functionalized with the chelator 1,4,7,10-tetraazacyclododecane-1,4,7,10-tetraacetic acid (DOTA–PEG–*b*–PBLG) [55]. The synthetic approach involved the ROP of BLG-NCA initiated by acetal–PEG–NH_2_, followed by termination of the amine group with *N*-succinimidyl propionate after the completion of the polymerization to afford the acetal-poly(ethylene glycol)–*b*–poly(*γ*-benzyl-l-glutamate)-propionate. ^1^H NMR resonance as well as SEC were used for the characterization of the polymer, which verified the expected molecular weight and very low polydispersity index. During the polymerization of BLG–NCA, FT-IR was used to monitor the monomer consumption. The acetal terminal groups were subsequently transformed to aldehyde by adding an aqueous HCl solution to the copolymer, followed by addition of NaOH solution to neutralize the pH. Subsequently, *p*–NH_2_–Bn–DOTA was added, and its amine group with the aldehyde of the copolymer. A Schiff base was obtained when DOTA had formed a covalent bound with the distal end of the PEG segment. The reduction of this imine to a secondary amine followed upon addition of a catalytic amount of NaBH_3_CN to give the final DOTA-PEG-*b*-PBLG block copolymer. ^1^H NMR and SEC analysis revealed that the polymer had the expected molecular weight and low polydispersity index, like its precursor.

The micelles were formed by adding dropwise MilliQ quality water in the solution of amphiphilic copolymers that was dissolved in DMAc, which was removed under vacuum. DLS was used to estimate the size of spherical micelles with a diameter of 27 nm. In-labeled micelles were prepared by incubation of the micelles in water by adding a solution of In. DLS showed that the diameter of In-containing micelles exhibited a diameter of 29 nm. In addition, Gd-labeled micelles were formed by adding GdCl_3_·6H_2_O in solution of the copolymer micelles in water. Dual labeled micelles containing both metals were prepared with a similar procedure. HPLC was used to verify their radiochemical purity and stability, ICP-MS was employed to determine the concentration of Gd loading and DLS indicated the micelle size of 28 nm diameter. In vivo studies in tumor mouse models showed that due to their small dimensions, micelles are suitable for the infiltration in the tumors with thick stroma, as is characteristic of scirrhous gastric cancer (SGC).

**Figure 15:**
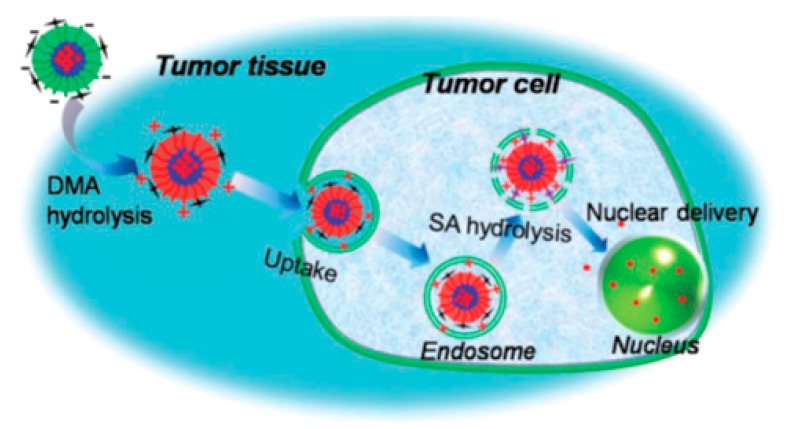
Mechanism for DOX delivery to tumors through nanoparticles [56].

Han et al. [56] synthesized dual-pH sensitive micelles from the self-assembly of the PLLeu–*b*–PLL(DMA)–Tat(SA), where the ε-amines of PLL have been amidated by 2,3-dimethylmaleic anhydride (DMA), and Tat peptide has been amidated with succinyl chloride (TAT(SA)). The synthetic approach involved the use of propargyl-amine as the initiator for the ROP of ZLL–NCA. Following completion of the polymerization, LLeu-NCA was added for the synthesis of the diblock polypeptide. Deprotection of the first block occurred by treatment with TFA and a 33% solution of HBr in acetic acid at 0 °C. Azido-terminated Tat peptide N_3_YGRKKRRQRRR was synthesized manually by the standard Fmoc SPPS protocol, followed by deprotection under alkaline conditions. The peptide was then amidated with succinyl chloride (N_3_-Tat(SA)), followed by the click reaction between PLL–*b*–PLLeu bearing a terminal triple bond and N_3_-Tat(SA) in the presence of catalytic amount of CuBr and tris(3-hydroxypropyltriazolylmethyl) amine. Finally, modification of lysine amino residues into *β-*carboxylic amide was carried out with the reaction of the obtained product with 2,3-dimethylmaleic anhydride to afford the dimethylacetamide protected PLL(DMA) (Figure 15).

For the preparation of the loaded nanoparticles, DOX∙HCl was dissolved in DMSO, and triethylamine was added along with the Tat modified polymer. PBS was added and dialysis took place several times until no DOX was found in the dialyzed water. The formed micelles were composed of a core of hydrophobic polyleucine blocks, while the hydrophilic shell was composed of polylysine blocks and Tat peptides were located in the outer corona. TEM and DLS revealed a spherical shape with diameter of about 20 nm. To prove the charge reversal behavior of the nanoparticles, zeta potential was measured at pH 7.4 and 6.5 and it was found that the zeta potential at 6.5 increased much more in comparison with that at pH 7.4. The in vitro drug release showed that DOX release was faster at pH 5, with 60% of the drug released at 48 h, while the release at 7.4 and 6.5 was similar, with 42% of the drug released after 48 h. The amides (Tat peptide as well as PLL(DMA)) followed stepwise hydrolysis, responding to acidic tumor extracellular environments (pH ≈ 6.5) and more acidic cell endo/lysosomes (pH ≈ 5.0), respectively, from which the amino groups and their original functions were recovered. In vitro studies also showed that the nanoparticles loaded with antitumor drugs featuring the Tat peptide, can convey cargos into the nuclei of HeLa cells very efficiently.

In 2016 Wu et al. [57] presented the synthesis of an innovative biocompatible surfactant based on Vitamin E-Oligo(methyl diglycol l-glutamate) (VEOEG). The synthesis of the polymer involved two steps. In the first, the hydroxyl group of Vitamin-E reacted with 4-nitrophenyl chloroformate in the presence of pyridine, followed by addition of ethylene diamine, to result in Vitamin E bearing a primary amine. Vitamin E-NH_2_ was used as the initiator for the ROP of methyl diglycol l-glutamate *N*-carboxyanhydride (EG_2_–Glu–NCA) to form the VEOEG surfactant. ^1^H NMR as well as MALDI-TOF analysis which revealed an Mn of 1695.1 g/mol, while SEC analysis showed a polydispersity of ~1.2. The preparation of poly(d,l-lactide-*co*-glycolide) nanoparticles (PLGL NPs) occurred by the nanoprecipitation method using VEOEG as a surfactant. The experimental route included the addition of PLGL solution in acetone to a VEOEG aqueous solution, evaporation of acetone after stirring, collection of the desired product by centrifugation and washing with deionized water. PLGL NPs exhibited a positive surface charge due to the terminal amino groups of VEOEG stabilizers located on the surface of NPs. The hydrodynamic diameter of NPs was about 135 nm as DLS analysis revealed, and the nanoparticles were spherical as confirmed by TEM. PLGL NPs were then coated with negatively charged hyaluronic acid (HA) and further stabilized by carbodiimide chemistry using EDC/NHS as coupling agents. TEM analysis confirmed the uniform size distribution of HA-PLGL NPs, and DLS analysis calculated a diameter of about 143 nm.

Finally, PTX was loaded into HA-PLGL NPs to form (PTX-HA-PLGL NPs) through the addition of acetone solution of PTX and PLGL to a water phase containing a predetermined quantity of VEOEG. DLS analysis estimated a mean size of 164 nm and TEM analysis showed a spherical morphology for the PTX–HA–PLGL NPs. DCC and DLE were measured by HPLC demonstrating that the encapsulating efficiency of the drug was 80.1%. The in vitro and in vivo studies revealed that PTX-HA-PLGL NPs had better anti-cancer effects in breast tumor cells than that of free paclitaxel.

The development of glycopolypeptide drug carriers can offer many advantages to drug delivery applications, such as targetability and stimuli-responsiveness, due to their biodegradable and biocompatible self-assembled structures. Lactobionolactone with the bioactive sugar moiety galactose can be used as a model ligand targeted to liver cancer. In 2017, Wang et al. [58] synthesized a lactobionolamidopropylazide (Lac-N_3_) initially using lactobionic acid which was converted to lactobionolectone by employing TFA as a catalyst, followed by addition of 3-azidopropylamine and a catalytic amount of DIPEA for the preparation of the final product. For the polypeptide synthesis, propargylamine was used as initiator for the sequential ROP of BLG-NCA and l-phenylalanine NCA. Removal of the benzyl protecting groups of l-glutamate was followed by treatment with Me_3_SiI. SEC and ^1^H-NMR analysis confirmed the successful polymerization. Conjugation of Lac-N_3_ to the alkyne group of the polypeptide was achieved via copper-catalyzed azide-alkyne cycloaddition.

The micelles were prepared through direct dissolution of the glycopolypeptides in a solution of pH 12, followed by sonication. The pH was then adjusted to 7.4 with 0.1M HCl. The glycopolypeptides and the polypeptides were self-assembled into spherical micelles as TEM, DLS and SAXS (Small Angle X-ray Scattering) revealed. The zeta potential values for the polypeptides showed a negative charged surface indicating that poly-l-glutamic acid is the outer chain, while the representative saccharide-conjugated micelles showed lower charges, an indication of the shield of the saccharide moiety. *Ricinus communis* agglutinin (RCA120) is a lectin, which is selective for the sugar moiety. The carbohydrate-lectin binding measurements suggested that galactose was on the surface of the micelle. Coil to helix/sheet secondary structure change was observed upon decreasing pH for polypeptides and glycopolypeptides. For the glycopolypeptides, an increase in the particle size and a decrease in surface negative charge were found at lower pH, while the polypeptides formed large aggregates at pH 6.0. DOX was loaded to the glycopolypeptide micelles through the nanoprecipitation methodology followed by centrifugation through a Vivaspin 6 centrifugal concentrator filter to remove free drug. In vitro DOX release studies were conducted and revealed the faster release of DOX at pH 5.5 than at pH 7.4. The glycopolypeptides bound to the cells through asialoglycoprotein receptor-mediated recognition. It was also found that the higher uptake of saccharideconjugated micelles could explain the higher drug release and cytotoxicity under pH-sensitive conditions.

## 9. Conclusions and Future Perspectives

Over the last 4 decades, tremendous advancements towards the discovery of novel drugs as well as their delivery systems have been reported for the treatment of cancers. The evolution of DDS has resulting in the development of novel materials with high specificity for controlled delivery. Drugs are selectively delivered to the cancer cells in a defined dose with minimum side effects. As a consequence, the development of novel molecules in combination with multifunctional delivery nanocarriers has resulted in once highly lethal diseases becoming chronic. Among more than 200 different cancers some, such as melanoma, are now chronic diseases due to novel carrier systems for known pharmaceuticals.

However, in spite of the important development of new materials, only limited clinical progress has been made to date for other forms of cancer, such as pancreatic and lung cancer. For these diseases with complex modes of action, more effective drugs as well as advanced drug carriers are required.

Among the nanoconstructs that have been developed as drug carriers, polypeptide-based micelles are promising for systemic administration. The presence of the polypeptide block affords them more precise structure and renders them more able to respond to a wide range of external stimuli due to the specific secondary structure of the polypeptide block. Due to their unique 3D structure (e.g., *α*-helix or *β*-sheet), polypeptides can mimic natural systems, thereby facilitating the development of smart nanodevices, which are able to carry a pharmaceutical compound and, by modifying their macroscopic properties, offer controlled delivery of their cargo.

In complex diseases such as cancer, very sophisticated nanocarriers should be designed to encapsulate a significant quantity of drugs, to bypass biological barriers with minimum cargo loss to effectively and selectively deliver the cargo to the desired pathological site. The collaboration of material as well as pharmaceutical scientists, biologists and clinical oncologists is imperative to produce efficient materials that possess advanced properties and required functionalities. Novel theories on cancer evolution and a better understanding of its biology have already provided insight into the cause of disease and its treatment. Furthermore, in our opinion, advancements in drug design and the development of multifunctional nanocarriers from the combination of well-defined macromolecular architectures and smart materials are the future for the effective treatment of many lethal diseases such as cancer. Table 1 summarizes the NCAs used, the corresponding polymers synthesized for the formation of the micelles, their stimuli as well as the loaded drug/gene cargo.

## Figures and Tables

**Table 1:** Summary of the micelles formed by polypeptide containing polymers synthesized via N-carboxy anhydrides to treat cancer. It is related to all reviewed papers.

NCA	Polymer	Stimuli	Loaded Drug/Other Cargo	Ref.
l-Leucine d-Leucine	PEG–*b*–P(l-Leu) PEG–*b*–P(d-Leu)	Unresponsive micelles	DOX	[8]
*γ*-Benzyl–l-glutamate	PBLG–*b*–PMPC	Unresponsive micelles	DOX	[9]
*β*-Benzyl–l-aspartate	PEG–*b*–P[Asp(ADR)]	pH	Adriamycin	[10]
*β*-Benzyl–l-aspartate	PAsp(OBzl)–*b*–PVP	pH	Prednisone acetate	[11]
*β*-Benzyl–l-aspartate	PEG–p(Asp–Hyd-X-Est-DEX) (‘X’ Indicates Ketonic Acids as Spacers)	pH	Dexamethasone	[12]
*ε*-Benzyloxycarbonyl-l-lysine, l-phenylalanine	PEG–*b*–PLL–*b*–PPhe	pH	DNA, DOX	[13]
l-Phenylalanine	Amphiphilic dendritic PLGA–*b*–PPhe	pH	DOX	[14]
*γ*-Benzyl–l-glutamate	alkyne-PAMA-g-PLGA	pH	DOX	[15]
*β*-Benzyl–l-aspartate	polyplex micelles: [PAsp(DET)] into (PEG)–*b*–PAsp(DET)	pH	–	[16]
*β*-Benzyl–l-aspartate	PEG–*b*–PHYD	pH	3-(3-pyridinyl)-1-(4-pyridinyl)-2-propen-1-one	[17]
*γ*-Benzyl–l-glutamate	mPEG–*b*–PLGA	pH	CDDP	[18]
*ε*-Benzyloxycarbonyl-l-lysine l-Leucine	PEG–*b*–PLL–*b*–PLLeu	pH	DTX siRNA	[19]
*ε*-Trifluoroacetyl-l-lysine *β*-Benzyl–l-aspartate	PEG–*b*–PLL–*b*–PAsp(DET–DN)	pH	siRNA	[20]
*γ*-Benzyl–l-glutamate l-phenylalanine	mPEG–*b*–P(Glu-*co*-Phe)	pH	DOX	[21]
Dinitrophenyl-protected histidine	Mixture of PEG*-b-*Phis and PEG–*b*–P(L–LA)–DTPA–Gd	pH	–	[22]
*ε*-Benzyloxycarbonyl–l-lysine	SPPCL–*b*–PZLLs	pH	–	[23]
*β*-Benzyl–l-aspartate	mPEG–*b*–PAsp	pH	DOX	[24]
*γ*-Benzyl–l-Glutamate	3-miktoarm star copolymer of Y-shaped (mPEG)_2_–PLGA	pH	PTX CDDP	[25]
Di-O,O’-acetyl–l-DOPA-*N*-carboxyanhydride	PEG-*b*-PDOPA	pH	DTX	[26]
*γ*-Camptothecin-glutamate	Graft copolymer of PEG and poly(*γ*-camptothecin-glutamate)	pH	Camptothecin	[27]
*γ*-Benzyl-l–Glutamate l-Phenylalanine	mPEG–*b*–P(Glu)–*b*–P(Phe)	pH	PTX CDDP	[28]
*β*-Benzyl–l-aspartate	PEG–*b*–P(Asp–Hyd–LEV–PTX) PEG–*b*–P(Asp–Hyd–LEV–RAP)	pH	PTX RAP	[29]
*Nε*-carbobenzoxy–l-lysine l-Phenylalanine	mPEG-g-PLL–*b*–Phe	pH	DOX	[30]
*N*-*im*-benzyl–l-histidine l-Phenylalanine	Phe–*b*–p(His)–*b*–PEG	pH	DOX, QUR	[31]
*γ*-Propargyl–l-glutamate	PLG40-g-P(MEOiMA)	Temperature	DOX	[32]
*N*-*im*-benzyl–l-histidine	p(NIPAM)–*b*–p(His)	Temperature	DOX	[33]
l-Leucine	mPEG–SS–*b*–PLLeu	Redox	DOX	[34]
3-Benzyloxycarbonyl–l-lysine	mPEG–SS–*b*–PZLL	Redox	DOX	[35]
*γ*-Benzyl–l-glutamate L-phenylalanine	PEG–*b*–PGlu(EDA–LA)-*b*-PPhe	Redox	DOX CDDP	[36]
*γ*-Benzyl–l-glutamate	mPEG–*b*–PBLG–SS–DTX	Redox	DTX	[37]
3-Benzyloxycarbonyl–l-lysine l-Leucine	PEG–*b*–PLL–*b*–PLLeu	Photo	–	[38]
S-(*O*-nitrobenzyl)-l–cysteine S-(*O*-nitrobenzyl)-d–cysteine	PC-*g*-PEG	Photo	DOX	[39]
*β*-Benzyl–l-aspartate	mPEG–*b*–PAsp(DA)	Multi	–	[40]
*γ*-Benzyl–l-glutamate l-phenylalanine l-cystine	PLGA–*b*–P(l-Phe-*co*-l-CS)	Multi	DOX RES	[41]
3-Benzyloxycarbonyl–l-lysine *β*-Benzyl–l-aspartate	PEG–*b*–PLL PEG–*b*–(PLL–Tyr_1_) PEG–*b*–PLL PNIPAM–*b*–PAsp PLAsp PLL	Multi	–	[42]
*β*-Benzyl–l-aspartate	POEGMA–*b*–PAsp(DIPEA)	Multi	DOX BODIPY-Br_2_	[43]
*γ*-Benzyl–l-glutamate	mPEG–SS–PNLG	Multi	DOX	[44]
Diethylene glycol mono-methyl ether-l-Glutamate	TMS-terminated and disulfide-bond-centered poly(EG_2_-l-glutamate)	Multi	DOX	[45]
Benzyl protected l-histidine l-phenylalanine	Poly(His-*co*-Phe)–*b*–PEG blended with PLLA–*b*–PEG–folate	Surface ligand	DOX	[46]
*γ*-Benzyl–l–glutamate	GA–PEG–*b*–PBLG	Surface ligand	DOX	[47]
*γ*-Benzyl–l–glutamate	GA–PEG–*b*–PBLG	Surface ligand	DOX	[48]
*β*-Benzyl–l–asparate	Folate conjugated heparin-PBLA folate conjugated PEG-*b*-PBLA	Surface ligand	–	[49]
*γ*-Benzyl–l–glutamate	R-PEG*-b-*PLGA(DOX) R is either cyclo(Arg–Gly-Asp-d-Phe–Cys) peptide or macrocyclic chelators NOTA	Surface ligand	DOX	[50]
*γ*-Propargyl–l-glutamate	PMLG–*b*–PLGA PGLG–*b*–PLGA	Surface ligand	DOX	[51]
*γ*-Benzyl–l-Glutamate	folate-decorated 3-arm star-block terpolymer [PGAH–*b*–(PDMAPMA-*g*-FA/PEG)]_3_	Surface ligand	DOX siRNA	[52]
3-Benzyloxycarbonyl–l-lysine l-phenylalanine	PEG–*b*–pLys–*b*–pPhe	Surface ligand	PTX	[53]
3-Benzyloxycarbonyl–l-lysine l-phenylalanine	PEG–*b*–pLys–*b*–pPhe DHAA–PEG–*b*–pLys–*b*–pPhe	Surface ligand	DOX	[54]
*γ*-Benzyl–l-Glutamate l-Leucine	DOTA–PEG–*b*–PBLG	Surface ligand	–	[55]
3-Benzyloxycarbonyl-l-lysine	PLLeu–*b*–PLL(DMA)–Tat(SA)	Surface ligand	DOX	[56]
Methyl diglycol l-glutamate	Vitamin E-Oligo(methyl diglycol l-glutamate)	Surface ligand	Hyaluronic acid PTX	[57]
*γ*-Benzyl–l-glutamate, l-phenylalanine	Lac–PGA–b–PPhe	Surface ligand	DOX	[58]

## Abbreviations

Acetal–PEG–*b*–PBLG	Acetal–poly(ethylene glycol)–*b*–poly(*γ*-benzyl l-glutamate)
(AC2)-DOPA–NCA	Di-*O*,*O*’-acetyl-l-l-3,4 dihydroxyphenylalanine-*N*-carboxyanhydride
ACN	Acetonitrile
ADR	Adriamycin
AFM	Atomic Force Microscopy
AIBN	2,2’-Azo-bis(isobutyronitrile)
Asp(OBzl)–NCA	*β*-Benzyl l-aspartate *N*-carboxy-anhydride
ATPEG	Poly(ethylene glycol)-bis-amine
ATRP	Atom Transfer Radical Polymerization
BLA	*β*-Benzyl–l-aspartate
BLG	*γ*-Benzyl–l-glutamate
BIED	Bis(*β*-isocyanatoethyl) disulfide
Bn-His	*N*-*im*-benzyl-l-histidine
Boc-l-His–(Bn)–OH	*N*-*α*-t-Butyloxycarbonyl-*N*-*im*-benzyl-l-histidine
BODIPY-Br_2_	3,7-Dibromo-2,8-di(4-methoxyphenyl)-11-trifluoromethyl-dithieno [2,3-b]-[3,2-g0-5,5-difluoro-5-bora-3a,4a-diaza-s-indacene
bpy	2,2’-Bipyridyl
CDDP, cisplatin	cis-Diamminedichlo-platinum
Ce 6	Clorin e6
CMC	Critical Micelle Concentrations
CP	Cloud Point
CR–CAs	Cancer–recognizable contrast agents
DCC	*N,N*’-Dicyclohexylcarbodiimide
DCM	Dicyclohexylcarbodiimide
DCU	Dicyclohexylurea
DDACT	S-1-dodecyl-S’-(*α,α’*-dimethyl-*α*’’-acetic acid) Trithiocarbonate
DEX	Dexamethasone
Dh	Hydrodynamic diameter
DHA	Dehydroascorbic acid
DHAA	Dehydroascorbic acid
DHPE	*N*-(5-dimethylaminoapthalene-1-sulfonyl)-1,2,-dihexadecanoyl-Sn-glycero-3-phosphoethanolamine
DIC	*N*,*N*’-diisopropylcarbodiimide
DIPEA	Diisopropylethylamine
DLC	Drug Loading Content
DLE	Drug Loading Efficiency
DLS	Dynamic Light Scattering
DMAc	*N*,*N*’-Dimethyl acetamide
DMAP	4-(Dimethylamino)pyridine
DMAPMA	*N*,*N*-dimethylaminopropyl methacrylamide
DMF	*N*,*N*-Dimethyloformamide
DMA	2,3-Dimethylmaleic anhydride
DMAP	4-Dimethylaminopyridine
DMPP	Dimethylphenylphosphine
DMSO	Dimethylosulfoxide
DOX	Doxorubicin
DOX·HCl	Doxorubicin hydrochloride
DOX-NP	DOX–nanoparticles
DOTA	1,4,7,10-tetraazacyclododecane–1,4,7,10–tetraacetic acid
DOTA/m	DOTA–functionalized polymeric micelles
DP	Degree of Polymerization
DTT	1,4-Dithiothreitol
DTDP	3,3′-Thiodipropionic acid
DTPA	Diethylene triamine pentaacetic
DTSSP	3,3’-dithiobis(sulfosuccinimidyl propionate)
DTX	Docetaxel
DTX–CLPMs	(DTX) loaded core-cross-linked polymer micelles
DTX–NPM	DTX-loaded non-crosslinked
EG_2_–Glu–NCA	ethyl diglycol L-glutamate *N*-carboxyanhydride
FA	Folic acid
FBS	Fetal bovine serum
Fmoc	Fluorenylmethyloxycarbonyl chloride
FT-IR	Fourier Transform Infrared Spectroscopy
GA	Glycyrrhetinic acid
GA–PEG–NH_2_	Glycyrrhetinic acid-modified poly(ethylene glycol)–bis-amine
GSH	Glutathione
HA	Hyaluronic acid
HBr-HAc	Hydrobromic acid-acetic acid
HCC	Hepatocellular carcinoma
HDI	Hexamethylenediisocyanate
^1^H NMR	Proton Nuclear Magnetic Resonance
HPLC	High Pressure Liquid Chromatography
Hyd	Hydrazine
ICG	Indocyanine green
IC_50_	The half maximal inhibitory concentration
ICP–MS	Inductively coupled plasma mass spectrometry
ICP–OES	Inductively coupled plasma–optical emission spectrometry
Ins-CAs	pH-insensitive cancer–recognizable contrast agents
LA	Lipoic acid
Lac-N_3_	Lactobionolamidopropylazide
LCST	Low critical solution temperature
LEV	Levulinic acid
L-Phe	l-phenylalanine
LLA	l-lactic acid
Lys(Z)	N^6^-carbobenzyloxy–l-lysine
MALDI-TOF MS	Matrix-assisted laser desorption/ionization Time of Flight Mass Spectrometry
MDR	Multidrug Resistance
MEO_2_MA	2-(2-methoxyethoxy)ethyl
MEO_3_MA	2-(2-(2-methoxyethoxy)ethoxy)ethyl methacrylate
mPEG–*b*–pAsp(DA)	Monomethoxy poly(ethylene glycol)–*b*–decylamine-grafted poly(l-aspartic acid)
mPEG–*b*–P(Glu-*co*-Phe)	Poly(ethyleneglycol)*–b–*poly(l-glutamic acid-*co*-l-phenylalanine)
mPEG–CHO	Methoxy poly(ethylene glycol)-aldehyde
mPEG–NH_2_	*α*-Μethoxy-ω-amine-poly (ethylene glycol)
(mPEG)_2_–*b*–PBG	(Μethoxy-poly(ethylene glycol))_2_-block-poly(g-benzyl-l-glutamate)
(mPEG)_2_–*b*–PGA	(Μethoxy-poly(ethylene glycol))_2_*–b–*poly(l-glutamic acid)
mPEG–PA	Methoxy-poly(ethylene glycol)-*block*-poly(aspartamide)
mPEG–PAlEA	Methoxy-poly(ethylene glycol)*–*poly(aldehyde ethyl aspartamide)
mPEG–PAmEA	Methoxy-poly(ethylene glycol)-block-poly(amino ethyl l-aspartamide)
mPEG–PBLG–SS–DTX	Conjugates poly(ethylene glycol)-poly(*γ*-benzyl l-glutamate)-disulfide-docetaxel
mPEG-*g*-PLL–*b*–Phe	Methoxypolyethylene glycol-graft-poly(l-lysine)-*block*-poly(l-phenylalanine)
mPEG–PPDA	Methoxy-poly(ethylene glycol)-*block*-poly(propanediol l-aspartamide)
Mn	Average molecular weight
MRI	Magnetic Resonance Imaging
MWCO	Molecular Weight Cut-Off
NaBH_3_CN	Sodium cyanoborohydride
NaN_3_	Sodium azide
NBS	*N*-Boc-serinol
NCA	*N*-carboxyanhydride
NHS	Hydroxysuccinimide
NIPAM	*N*-Isopropylacrylamide
NIR	Near Infrared
NIRF	Near-infrared fluorescent
NMAm	*N*-methylol acrylamide
NMP	*N*-methyl pyrrolidone
NOTA	1,4,7-Triazacyclononane-*N*,*N*’,*N*’’-triacetic acid
NPs	Nanoparticles
N_3_-PEG-NH_2_	*α*-azide-ω-amino-poly(ethylene glycol)
PA	Photoacoustic
PAMA	Poly(2-aminoethyl methacrylate)
(PGAH–*b*–PDMAPMA)_3_-*g*-PEG	[poly(l-glutamic acid *γ*-hydrazide)–*b*–poly(*N*,*N*-dimethylaminopropyl methacrylamide]_3_-*g*-poly(ethylene glycol)
PBS	Phosphate-buffered saline
PBLG–*b*–PMPC	Poly(*γ*-benzyl–l-glutamate)–block–poly(2-Methacryloyloxyethyl phosphorylcholine)
PC-*g*-PEG	Poly(cysteine)-*g*-poly(ethylene glycol)
PDI	Polydispersity
PDT	Photodynamic therapy
PEG	Poly(ethylene glycol)
PEG–PAA	Poly(ethylene glycol)*–b–*poly(amino acids)
PEG–*b*–P(d-Leu)	Poly(ethyleneglycol)*–b–*poly(d-Leucine)
PEG*–*p(HYD)	Poly(ethyleneglycol)–*b*–poly(aspartate hydrazide)
PEG*–b–*p(Asp)	Poly(ethylene glycol)–*b*–poly(*β*-benzyl-l-aspartate)
PEG*–b–*PBLG	Poly(ethylene glycol)–*b*–poly(*γ*-benzyl-l-glutamate)
PEG*–b–*PLL*–b–*PLLeu	Poly(ethylene glycol)–*b*–poly(l-lysine)-*b*-poly(l-leucine)
PEG*–b–*p(l-His)	Poly(ethylene glycol)–*b*–poly(l-histidine)
PEG–*b*–p(l–LA)–DTPA–Gd	Poly(ethylene glycol)–*b*–poly(l-lactic acid)- Diethylenetriaminopentaacetic acid dianhydride- gadolinium chelate
PEG–*b*–P(l-Leu)	Poly(ethylene glycol)–*b*–poly(l-Leucine)
PEG–*b*–PGlu(EDA–LA)–*b*–PPhe	Poly(ethylene glycol)–*b*–poly(*γ*-benzyl–l-glutamate)–*b*–poly(l-phenylalanine)
PGLG	Poly(galactosylated) l-glutamate
Phe	Phenylalanine
PLGA	Poly(d,l-lactide-*co*-glycolide)
PLGA–b–PPhe	Poly(glutamic acid)–*b*–polyphenylalanine
PLG NCA	*γ*-Propargyl-l-glutamate methacrylate
PLGA	Poly(d,l-lactide-*co*-glycolide)
PLLZ	Poly(Nε-carbobenzyloxy-l-lysine)
PMLG	Poly(oligo(ethylene glycol)l-glutamate
PNBC	poly(*S*-(*O*-nitrobenzyl)-d,l-cysteine)
PPLG	Poly(*γ*-propargyl–l-glutamate)
[p(NIPAM)–*b*–p(His)]	Poly(*N*-isopropylacrylamide)–*b*–poly(l-histidine)
POEGMA	Poly[oligo(ethylene glycol) methacrylate]
PS	Photosensitizer
PTT	Photothermal therapy
PTX	Paclitaxel
QUR	Quercetin
RAFT	Reversible addition fragmentation chain transfer polymerization
RAP	Rapamycin
RES	Resveratrol
ROP	Ring Opening Polymerization
RP–HPLC	Reversed-phase high pressure liquid chromatography
SA	Succinyl chloride
SCLM	Shell cross-linking micelles
SEC	Size exclusion chromatography
SGC	Scirrhous gastric cancer
siRNA	Small interfering RNA
SPPCL–*b*–PZLLs	Poly(ε-caprolactone)–*b*–poly(ε-benzyloxycarbonyl–l-lysine)
TAEA	Tri(2-aminoethyl) amine
TEA	Triethylamine
TEM	Transmission electron microscopy
TFA	Trifluoroacetic acid
THF	Tetrahydrofuran
VE	Vitamin E
VE-NH_2_	Vitamin E-ethylenediamine
VEOEG	Vitamin E-Oligo(methyl diglycol l-glutamate)
Z-L-Lys NCA	Nε-benzyloxycarbonyl–l-lysine *N*-carboxyanhydride
3PO	3-(3-Pyridinyl)-1-(4-pyridinyl)-2-propen-1-one
4-NC	4-Nitrophenyl chloroformate
*γ*-Bzl–l-Glu-NCA	5-Benzyl-l-glutamate-*N*-carboxyanhydride
